# NMR analyses on *N*-hydroxymethylated nucleobases – implications for formaldehyde toxicity and nucleic acid demethylases†
†Electronic supplementary information (ESI) available. See DOI: 10.1039/c8ob00734a


**DOI:** 10.1039/c8ob00734a

**Published:** 2018-05-16

**Authors:** S. Shishodia, D. Zhang, A. H. El-Sagheer, T. Brown, T. D. W. Claridge, C. J. Schofield, R. J. Hopkinson

**Affiliations:** a Chemistry Research Laboratory , 12 Mansfield Road , Oxford , OX1 3TA , UK; b Leicester Institute of Structural and Chemical Biology and Department of Chemistry , University of Leicester , Henry Wellcome Building , Lancaster Road , Leicester , LE1 7RH , UK . Email: richard.hopkinson@leicester.ac.uk; c Chemistry Branch, Department of Science and Mathematics , Faculty of Petroleum and Mining Engineering , Suez University , 43721 Suez , Egypt

## Abstract

Formaldehyde is produced in cells by enzyme-catalysed demethylation reactions, including those occurring on *N*-methylated nucleic acids. Formaldehyde reacts with nucleobases to form *N*-hydroxymethylated adducts that may contribute to its toxicity/carcinogenicity when added exogenously, but the chemistry of these reactions has been incompletely defined. We report NMR studies on the reactions of formaldehyde with canonical/modified nucleobases. The results reveal that hydroxymethyl hemiaminals on endocyclic nitrogens, as observed with thymidine and uridine monophosphates, are faster to form than equivalent hemiaminals on exocyclic nitrogens; however, the exocyclic adducts, as formed with adenine, guanine and cytosine, are more stable in solution. Nucleic acid demethylase (FTO)-catalysed hydroxylation of (6-methyl)adenosine results in (6-hydroxymethyl)adenosine as the major observed product; by contrast no evidence for a stable 3-hydroxymethyl adduct was accrued with FTO-catalysed oxidation of (3-methyl)thymidine. Collectively, our results imply *N*-hydroxymethyled adducts of nucleic acid bases, formed either by reactions with formaldehyde or *via* demethylase catalysis, have substantially different stabilities, with some being sufficiently stable to have functional roles in disease or the regulation of nucleic acid/nucleobase activity.

## Abstract 2

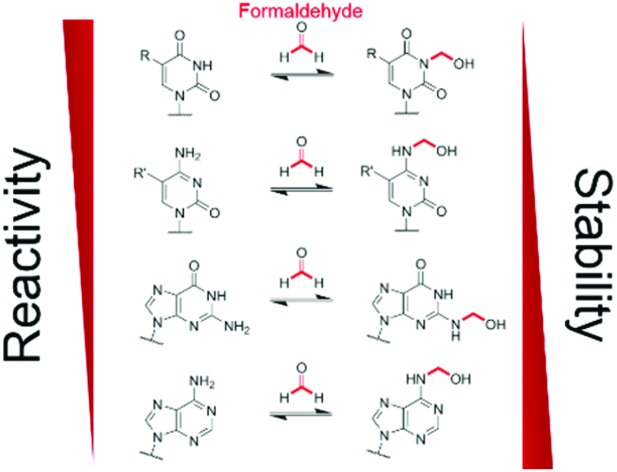
NMR studies reveal that formaldehyde, a toxic pollutant and metabolite, reacts with nucleotides to form *N*-hydroxymethylated adducts of varying stabilities.

## Introduction

Formaldehyde, the simplest aldehyde, exists principally in its hydrated form in water and is toxic and carcinogenic to humans above threshold levels.^1^ Acute exposure to HCHO can lead to pulmonary oedema,^2^ renal failure^3^ and inflammation,^4^ while chronic HCHO exposure correlates with increased incidence of cancer.^5^ HCHO toxicity/carcinogenicity is likely a consequence of its reactions with biomolecules; HCHO is reported to cross-link DNA,^6^ while reactions with some proteins are proposed to induce sensory responses^7^,^8^ and protein aggregation.^9^,^10^

In addition to exogenous sources, *e.g.* from plastics,^1^ food^11^,^12^ and cosmetics,^13^,^14^ HCHO is produced endogenously during folate oxidation^15^ and by the action of enzymes including semicarbazide-sensitive amine oxidase.^16^ HCHO is also produced by the action of *N*-methyl group demethylases acting in both DNA repair and chromatin regulation.^17^ Endogenous HCHO levels are difficult to quantify, although reports estimate concentrations in the mid to high micromolar range;^12^,^15^,^18^–^21^ there is also potential for relatively high localised HCHO concentrations, *e.g.* in cell nuclei, where *N*-methyl demethylases are present. The physiological implications of endogenous HCHO production are poorly appreciated, although is emerging evidence that it is a carbon source for C1 metabolism.^15^ Cellular and mouse studies have also revealed a correlation between endogenous HCHO and toxicity in Fanconi Anaemia.^22^,^23^ HCHO is also proposed as a prebiotic precursor.^24^,^25^

**Fig. 1:**
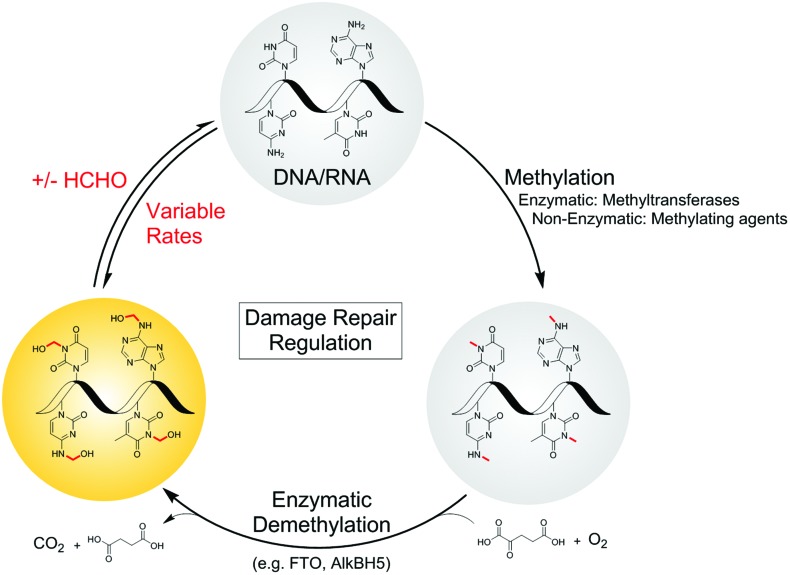
Formation of *N*-hydroxymethyl adducts on nucleic acids can occur *via* both enzymatic and non-enzymatic pathways. The nucleophilic nitrogens of nucleic acid bases react in non-enzyme-catalysed reactions with electrophiles such as formaldehyde (forming *N*-hydroxymethyl adducts) and methylating agents. *N*-Methylation is also catalysed at some sites by methyltransferases. Although not reported, there is potential for enzyme-catalysed reactions of nucleobases and formaldehyde. Many *N*-methylated nucleobases are substrates for 2-oxoglutarate (2OG)-, oxygen-, and ferrous iron-dependent nucleic acid demethylases, which catalyse demethylation *via* an oxidative mechanism involving formation of *N*-hydroxymethyl intermediates, which in some cases could be considered as products. As shown in the present work, there are considerable variations in the stabilities of the hydroxymethyl adducts.

Defining the chemistry underpinning HCHO-driven toxicity/carcinogenicity is challenging due to the likely reversible nature of many HCHO-biomolecule adducts.^26^,^27^ Hence it is important to define in detail the nature of HCHO adducts with isolated components, including nucleobases. Pioneering work by McGhee and von Hippel defined hemiaminal adducts produced by reaction of the canonical nucleobases with HCHO and revealed the potential of HCHO adducts to modulate base-pairing (hydroxymethylated adenine can base-pair with thymine, but in a less stable manner than unmodified adenine).^28^,^29^ The exocyclic amino groups of adenine, guanine and cytosine react to give hemiaminal adducts, as do the endocyclic imino groups of thymine and uracil. These findings are consistent with mass spectrometric (MS) studies on oligonucleotides, which identified nucleobase *N*-methylation on treatment with HCHO and NaCNBH_3_.^30^,^31^ Cross-linking between nucleobases after treatment of DNA with HCHO has also been observed;^32^,^33^ recent studies by Swenberg and co-workers have supplied evidence for cross-linked adducts between guanine and nucleophilic amino acids, involving aminal and hemithioaminal linkages.^34^–^36^ Interestingly, many of the observed nucleobase adducts are structurally analogous to the proposed hemiaminal intermediates produced during enzyme-catalysed *N*-methyl group demethylation (Fig. 1).^17^

We are interested in understanding how the physiological effects of HCHO arise from its reactions, and in testing the hypothesis that nucleic acid-HCHO adducts have functional roles in biology and disease. We report studies on the reactions of HCHO with both canonical and non-canonical nucleotides/nucleosides, some which have emerged since the work of McGhee and von Hippel,^28^,^37^ using NMR to determine their relative rates of formation and stability. To highlight the relevance of the results to biology, we also profiled the reactions of *N*-methylated nucleosides with the human nucleic acid demethylase FTO. Studies on FTO catalysis reveal that the stabilities of the hemiaminal products depend on the position of *N*-methyl group on the substrate; the major product of FTO-catalysed oxidation of (6-methyl)adenosine was the hemiaminal, whereas oxidation of (3-methyl)thymidine revealed only the demethylated product and HCHO. These observations reveal the potential for site-specific accumulation of *N*-hydroxymethyl adducts in nucleic acids. The combined studies suggest different hemiaminal adducts predominate under different conditions, and imply that the outcomes of nucleic acid *N*-methyl group oxidation may be more complex than previously perceived.

## Results and discussion

### HCHO reactions with canonical nucleotides

**Fig. 2:**
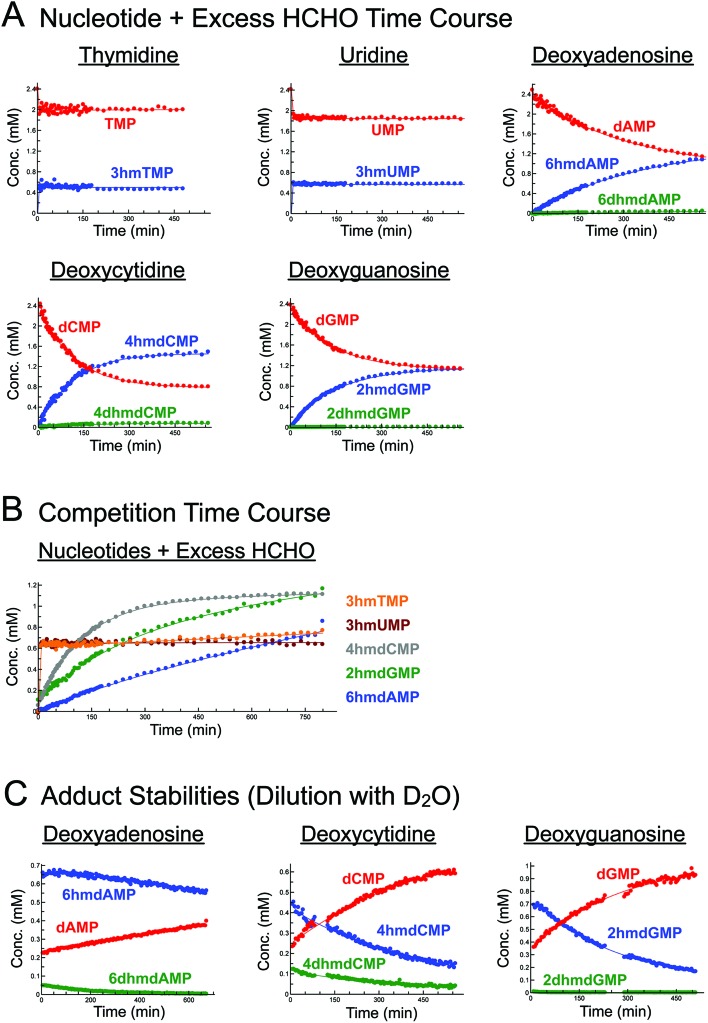
Canonical nucleotides react with HCHO at either exo- or endocyclic nitrogens and at different rates to form *N*-hydroxymethyl adducts. (A) ^1^H NMR time course analyses of incubations of canonical nucleotides with HCHO. Five canonical nucleotide monophosphates (dAMP, dCMP, dGMP, TMP, and UMP) were incubated with 53 eq. of HCHO in D_2_O at pD 7.5, and the reactions were monitored over time using ^1^H NMR (final nucleotide concentration = 2.4 mM). The pyrimidines TMP and UMP react *via* their endocyclic N-3 nitrogens to form 3hmTMP and 3hmUMP, respectively (blue). dAMP, dCMP and dGMP react *via* their exocyclic N-6, N-4 and N-2 nitrogens respectively to form 6hmdAMP, 4hmdCMP, and 2hmdGMP as major products (blue). Under the tested conditions, evidence for low-level formation of the dihydroxymethylated adducts 6dhmdAMP and 2dhmdGMP was also accrued (green). (B) ^1^H NMR time course analysis of a reaction mixture containing dAMP, dCMP, dGMP, TMP and UMP (all at 2.2 mM) and HCHO (58 eq.) in D_2_O at pD 7.5. The fastest adducts to form were 3hmTMP and 3hmUMP, formed *via* reactions of HCHO with TMP and UMP respectively. The other observed adducts (6hmdAMP, 4hmdCMP and 2hmdGMP), formed *via* reactions of HCHO with exocyclic nitrogens on dAMP, dCMP and dGMP respectively, were slower to form, but showed time-dependent increases in concentration. Of these three products, 4hmdCMP was fastest to form, followed by 2hmdGMP and 6hmdAMP respectively. Only the major adducts for each nucleotide are shown. (C) ^1^H NMR time course analyses of incubations of dAMP, dCMP and dGMP with HCHO, after two weeks reaction and subsequent dilution with D_2_O (25-fold). In all cases, the concentrations of the *N*-hydroxymethyl adducts decreased in a time-dependent manner after dilution, revealing non-immediate adjustments to new equilibrium positions. Of the three nucleotides, degradation of adducts derived from dAMP (6hmdAMP and 6dhmdAMP) were the slowest to degrade, revealing their relative stabilities compared to the other adducts. Upon dilution, 3hmTMP and 3hmUMP immediately decreased in concentration, indicating a fast dynamic equilibrium between the adducts and free nucleotides (and HCHO).

Initially, we incubated excess HCHO (53-fold) with the five canonical DNA/RNA nucleotide monophosphates deoxyadenosine monophosphate (dAMP), deoxycytidine monophosphate (dCMP), deoxyguanosine monophosphate (dGMP), thymidine monophosphate (TMP), and uridine monophosphate (UMP, all at 2.4 mM) at pD 6, pD 7.5 and pD 9, monitoring reaction over 5–8 hours by ^1^H NMR (Fig. 2A, S1–S3, S5 and S6†). A sample simultaneously containing all five nucleotides (each at 2.2 mM) and HCHO (58 equivalents) was also analysed (800 minutes at pD 7.5) to robustly compare their relative reaction rates (Fig. 2B).

As reported by McGhee and von Hippel, we observed reactions between HCHO and all five nucleotides (Scheme 2 and Fig. 2). With TMP, the major product was observed at the first time-point (7 minutes after mixing, Fig. 2A and Fig. S1†) and was present at constant concentration during the analysis (0.4 mM at pD 7.5 over 6 hours, Fig. 2A). The product was characterised as (3-hydroxymethyl)thymidine monophosphate (3hmTMP), on the basis of ^1^H and ^13^C NMR assignments, indicating that HCHO preferentially reacts on the N-3 nitrogen of TMP. While this endocyclic product is rapidly produced, its concentration (0.4 mM at pD 7.5) was low relative to unreacted TMP (2 mM at pD 7.5). Formation of 3hmTMP was too fast to determine its initial formation rates at pDs 7.5 and 9; however, the reaction was slower at pD 6 (3hmTMP formation rate = 0.3 μM s^–1^). On dilution of the reaction mixtures with D_2_O (2- to 25-fold), the concentration of 3hmTMP rapidly decreased from the first time-point to reform TMP, implying 3hmTMP is in dynamic equilibrium with TMP. Rapid fragmentation of 3hmTMP to TMP was observed on addition of the HCHO ‘scavenger’ dimedone.^39^ Identical behaviour (within our limits of detection) was observed with mixtures of HCHO and the RNA nucleotide uridine monosphosphate (UMP), also forming the endocyclic adduct (3-hydroxymethyl)uridine monophosphate (3hmUMP, formation rate at pD 6 = 0.783 μM s^–1^, Fig. 2A and Fig. S2†).

**Scheme 1:**
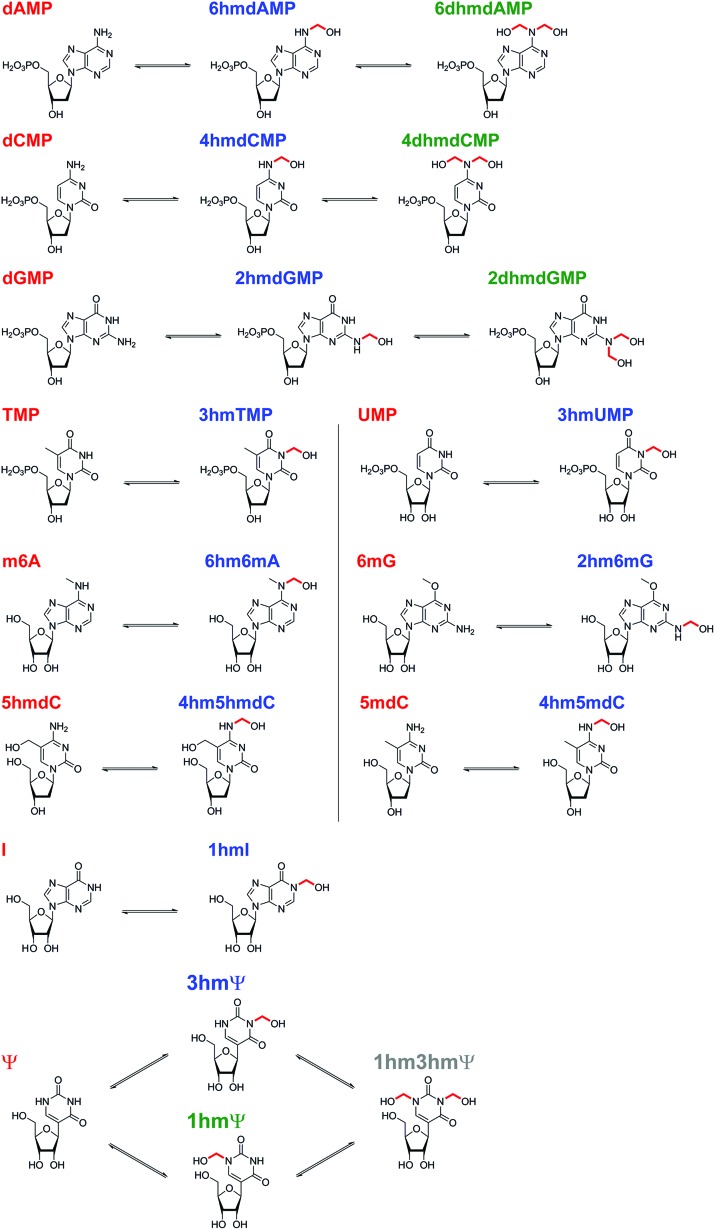
Summary of *N*-hydroxymethyl adducts observed upon treatment of nucleotides with HCHO. Five canonical nucleotides were tested for reaction with HCHO; dAMP, dCMP, dGMP, TMP and UMP. Seven modified nucleotides were also tested; 1 mA, m6A, 6mG, 5hmdC, 5mdC, I, and Ψ. Reactions were observed with all nucleotides (note: reaction with 1 mA was too low-level for NMR characterisation). The rates of reaction, however, vary significantly.

The other canonical nucleotides reacted with HCHO *via* their exocyclic nitrogens (Scheme 1). dCMP preferentially reacted with HCHO *via* its exocyclic N-4 nitrogen forming (4-hydroxymethyl)deoxycytidine monophosphate (4hmdCMP) as the major product (Fig. 2A and Fig. S3†). This product, which was observed as two conformational isomers (Fig. S3†), formed more slowly than 3hmTMP or 3hmUMP (formation rate at pD 7.5 = 0.2 μM s^–1^, Fig. 2B), but reached a higher maximal concentration (1.3 mM) after 350 minutes at pD 7.5 (Fig. 2A). No significant differences in the reaction profile was observed at pDs 6, 7.5 and 9 (formation rates = 0.117 μM s^–1^ and 0.133 μM s^–1^ at pDs 6 and 9 respectively, Fig. 2A and Fig. S3B/C†). Two additional ^1^H-resonances were observed at low levels (at *δ*_H_ 4.97 ppm, *δ*_H_ 6.35 ppm and *δ*_H_ 8.16 ppm at pD 7.5), which were assigned as the *N*-hydroxymethyl and aromatic protons of (4,4-dihydroxymethyl)deoxycytidine monophosphate (4dhmdCMP). This assignment, as well as that of 4hmdCMP, was supported by MS analyses on the reaction mixture at pD 7.5, which revealed the presence of species with masses expected for dCMP (308 Da, [M + H]^+^), a monohydroxymethylated adduct (338 Da, [M + H]^+^) and a dihydroxymethylated adduct (366 Da, [M – H]^–^) respectively. On D_2_O dilution (2- to 25-fold), both 4hmdCMP and 4dhmdCMP degraded to free HCHO and dCMP, demonstrating dynamic equilibrium; however, the rates of degradation (0.133 μM s^–1^ for 4hmdCMP, 0.003 μM s^–1^ for 4dhmdCMP after 25-fold dilution) were significantly slower than the near-instantaneous degradation observed for 3hmTMP (Fig. 2C and Fig. S4A/B†).

dGMP reacted with HCHO to form the exocyclic adduct (2-hydroxymethyl)deoxyguanosine monophosphate (2hmdGMP) as the major product (Fig. 2A/B and Fig. S5†). The emergence of a ^1^H-resonance at *δ*_H_ 5.07 ppm, likely corresponding to a hemiaminal, implied formation of a second adduct, although the maximal concentration of this species (0.06 mM after 360 minutes at pD 9, formation rate = 0.005 μM s^–1^, Fig. S5C†) was significantly lower than that of 2hmdGMP (1.2 mM after 360 minutes at pD 9, formation rate = 0.117 μM s^–1^, Fig. S5C†). ^1^H–^13^C-HMBC analyses revealed correlations between this resonance and both the C-2 carbon (at *δ*_C_ 154 ppm) and to itself (at *δ*_C_ 72 ppm); the species was therefore (provisionally) assigned as the *N*2-dihydroxymethylated adduct (2,2-dihydroxymethyl)deoxyguanosine monophosphate (2dhmdGMP). As with dCMP, no significant changes in adduct formation rates were observed across the 3 pD's tested (formation rates for 2hmdGMP = 0.1 μM s^–1^ and 0.117 μM s^–1^ at pDs 6 and 7.5 respectively, formation rates for 2dhmdGMP were too low to be accurately determined at pDs 6 and 7.5, Fig. 2A and Fig. S5B/C†). On dilution of the reaction mixtures with D_2_O (2- to 25-fold), both adducts degraded to free HCHO and dGMP; the degradation rates of 2hmdGMP and 2dhmdGMP (0.033 μM s^–1^ and 0.003 μM s^–1^ respectively at pD 7.5 after 25-fold dilution) were comparable to those observed for 4hmdCMP and 4dhmdCMP (Fig. 2C and Fig. S4E/F†).

dAMP preferentially reacted with HCHO *via* its exocyclic N-6 nitrogen, but reacted more slowly than the other canonical nucleotides (Fig. 2A/B and Fig. S6†). One major and one minor product were detected; the major product, (6-hydroxymethyl)deoxyadenosine monophosphate (6hmdAMP), was observed to increase in concentration throughout the analyses, reaching a maximum of 1.2 mM at pD 9 (formation rates = 0.067 μM s^–1^, 0.05 μM s^–1^ and 0.05 μM s^–1^ at pDs 6, 7.5, and 9 respectively, Fig. 2A and Fig. S6B/C†). The minor product was provisionally assigned as *N*6-dihydroxymethylated (6,6-dihydroxymethyl)deoxyadenosine monophosphate (6dhmdAMP) on the basis of ^1^H–^13^C-HMBC analyses, which revealed correlations between the hemiaminal resonance (at *δ*_H_ 5.35 ppm and *δ*_C_ 72 ppm) and both the C-6 carbon (at *δ*_C_ 154 ppm) and itself. Formation rates for this adduct were too low to be accurately determined (<0.003 μM s^–1^). Analyses with adenosine AMP, ADP and ATP revealed similar reactions to those of dAMP, suggesting that the outcomes of reactions with HCHO are not significantly affected under the tested conditions by the presence of a 2′ alcohol or additional phosphate groups (Fig. S7†). Interestingly, the dAMP adducts were apparently more stable than the adducts formed from the other deoxynucleotides, as evidenced by D_2_O dilution studies; under the conditions tested, 6hmdAMP was present in high concentrations for over 10 hours (degradation rate = 0.003 μM s^–1^ at pD 7.5 after 25-fold dilution, Fig. 2C and Fig. S4C/D†).

NMR analyses were then undertaken on mixtures containing the canonical nucleotides and 4 equivalents of HCHO (Fig. S8†). Formation of the dihydroxymethylated adducts (4dhmdCMP, 2dhmdGMP and 6dhmdAMP) was not observed; however, the monohydroxymethylated adducts 3hmTMP, 3hmUMP, 4hmdCMP, 2hmdGMP and 6hmdAMP were observed, suggesting these adducts might be more biologically relevant (note: physiological HCHO concentrations, especially at the sub-cellular level, are currently unclear). While the formation rates for 4hmdCMP, 2hmdGMP and 6hmdAMP were markedly slower than in the mixtures with excess HCHO (Fig. S8†), analyses of the mixtures containing TMP and UMP revealed rapid equilibration (*i.e.* equilibrium was reached prior to the first NMR analysis), although the concentrations of 3hmTMP and 3hmUMP were low (both 0.04 mM).

Temperature dependence studies on the reactions of HCHO with the canonical nucleotides were then carried out. Formation of adducts from mixtures of excess HCHO with the five canonical nucleotides (2.4 mM) was monitored over one hour at 10 °C (Fig. S9†). As expected, the formation rates for all adducts were slower at 10 °C than at 25 °C; formation of 3hmTMP was sufficiently slow to enable a provisional formation rate to be determined (0.617 μM s^–1^). Formation of 3hmUMP was largely complete before the first NMR analysis. Analysis of mixtures of HCHO with dAMP, dCMP and dGMP over one hour at 15, 20, 25, and 37 °C revealed a near-linear dependence on adduct formation rates with temperature (Fig. S10†). For the *N*-monohydroxymethylated adducts, formation of 6hmdAMP was slowest, followed by formation of 2hmdGMP and 4hmdCMP respectively (Fig. S10†). A similar general trend was observed for the dihydroxymethylated adducts, although their low levels in many of the samples precluded accurate determination of formation rates.

1-Dimensional Exchange Spectroscopy (1D-EXSY) was then carried out on the reactions to investigate exchange rates between the nucleotides and their HCHO-derived adducts. EXSY, which utilises the same pulse sequences used to identify nuclear Overhauser effect (nOe) correlations, can be applied to identify correlations between species in chemical exchange; the exchange-peak intensity growth rates at different mixing times are proportional to the exchange rates.^40^–^43^ The intensities of the *N*-hydroxymethyl ^1^H resonances of the nucleotide-HCHO adducts (*δ*_H_ 5–6 ppm) were determined after pre-irradiation of the ^1^H-resonance of hydrated HCHO (*δ*_H_ 4.74 ppm at 25 °C). No *N*-hydroxymethyl resonances were observed with dAMP or dGMP at 25 °C or 45 °C (dCMP was not studied as the chemical shifts of the ^1^H resonances for 4hmdCMP and 4dhmdCMP were too close to the HCHO ^1^H-resonance).

By contrast, 1D-EXSY analyses with TMP and UMP revealed exchange between 3hmTMP/TMP and 3hmUMP/UMP, respectively (Fig. S11†), implying the establishment of fast dynamic equilibria. Initial formation rates for 3hmTMP and 3hmUMP were calculated by measuring the intensities of the *N*-hydroxymethyl ^1^H-resonances at different mixing times (0.01–1.5 s, for details see ESI†). The results reveal that formation of 3hmTMP and 3hmUMP is significantly faster than for the adducts of dAMP, dGMP and dCMP (initial formation rates at 25 °C: 65 μM s^–1^ and 97 μM s^–1^, respectively). Plotting the second order rate constants for adduct formation as a function of temperature gave activation energies of 103.7 kJ mol^–1^ and 86.6 kJ mol^–1^ for 3hmTMP and 3hmUMP, respectively (Fig. S11†).

Overall, the NMR studies with canonical nucleotides reveal formation of distinct *N*-hydroxymethylated adducts with different formation rates and stabilities. With the exception of TMP and UMP, NMR-observable HCHO reactions occur on the exocyclic nitrogens, with the adducts of dAMP being most stable; TMP and UMP react fastest but their adducts are in dynamic exchange with the free nucleotides.

### Reactions of HCHO with modified nucleosides

**Fig. 3:**
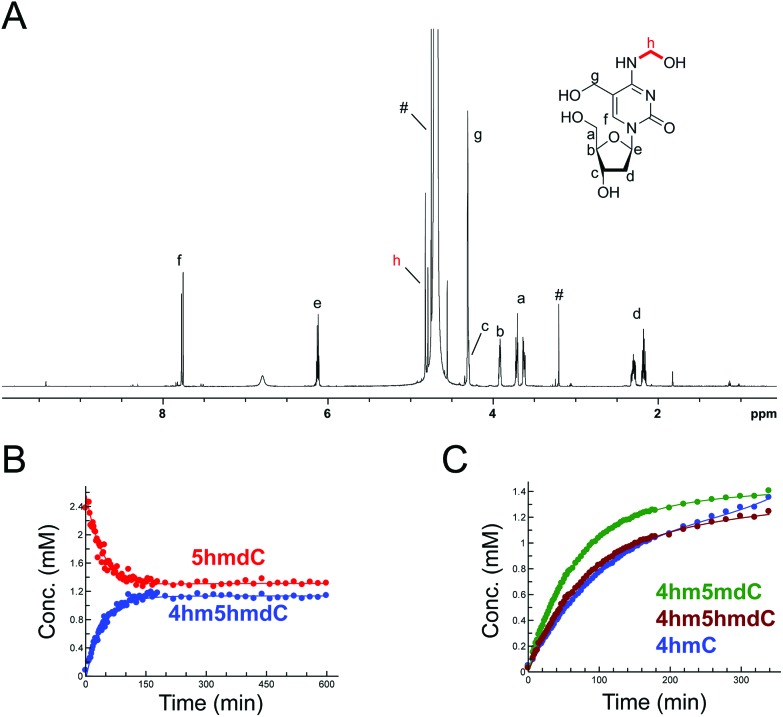
HCHO reacts with modified cytosine nucleosides to give *N*-hydroxymethyl adducts. (A) ^1^H NMR spectrum of 4hm5hmdC, formed after reaction of 5hmdC with HCHO. ^1^H-resonances for 4hm5hmdC are highlighted (note: unreacted 5hmdC is also present in the mixture – its ^1^H-resonances overlap with those of 4hm5hmdC). ^1^H-resonances marked with # are derived from HCHO. (B) ^1^H NMR time course analysis of a reaction mixture containing 5hmdC (2.4 mM) and HCHO (53 eq.) in D_2_O at pD 7.5. Time-dependent formation of 4hm5hmdC is observed, with concomitant decrease in 5hmdC. (C) ^1^H NMR time course analysis of a reaction mixture containing cytidine (C), 5mdC and 5hmdC (all 2.4 mM) and HCHO (53 eq.) at pD 7.5. d5mC reacted mostly efficiently with HCHO, forming 4hm5mdC (green), followed by 5hmdC (forming 4hm5hmdC, maroon) and C, forming 4hmC (blue).

The reactions of HCHO with non-canonical bases, *i.e.* (5-methyl)deoxycytidine (5mdC), (5-hydroxymethyl)deoxycytidine (5hmdC), (1-methyl)adenosine (1 mA), (6-methyl)adenosine (m6A), (6-methyl)guanosine (6mG), inosine (I), and pseudouridine (Ψ) were then analysed. As with the canonical nucleotides, mixtures containing the nucleoside with a 53-fold excess of HCHO in D_2_O at pD 7.5, were analysed over 600–800 min. No/trace reaction was observed between HCHO and 1 mA; however, NMR-observable products were detected in the samples with 5mdC, 5hmdC, m6A, 6mG, I and Ψ. Of these nucleotides, m6A reacted least efficiently with HCHO (Fig. S12†); formation of the only observed product, (6-hydroxymethyl-6-methyl)adenosine (6hm6mA), was slower than 6hmdAMP formation (formation rate of 6hm6 mA = 0.033 μM s^–1^). Both 5mdC and 5hmdC react with HCHO *via* their exocyclic N-4 nitrogens (as for dCMP) to form *N*4-hydroxymethylated adducts as major products (4-hydroxymethyl-5-methyl)deoxycytidine (4hm5mdC) and (4-hydroxymethyl-5-hydroxymethyl)deoxycytidine (4hm5hmdC), respectively (Fig. 3 and Fig. S13†). Low-level resonances were also observed but were too weak for assignment. A competition experiment, where cytidine (C), 5mdC and 5hmdC were treated together with HCHO, revealed that the formation rate for 4hm5mdC was faster than that observed for 4hm5hmdC and (4-hydroxymethyl)cytidine (4hmC); with the exception of 3hmTMP and 3hmUMP, 4hm5mdC was the fastest to form of all the observed adducts (formation rate = 0.233 μM s^–1^, Fig. 3C). 6mG reacted with HCHO in a similar manner to dGMP, forming (2-hydroxymethyl-6-methyl)guanosine (2hm6mG, formation rate = 0.05 μM s^–1^, Fig. S14†). Other low-level resonances were also observed in the spectra but were too weak to fully assign; they may reflect low level formation of dihydroxymethylated adducts.

**Fig. 4:**
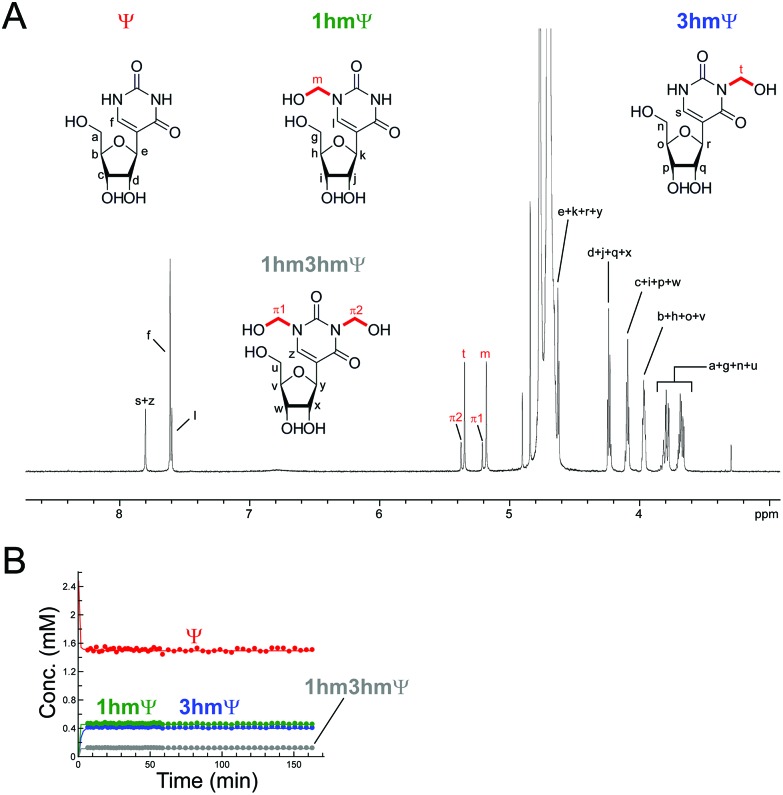
HCHO reacts with Ψ to give three hydroxymethylated adducts. (A) ^1^H NMR spectrum of a reaction mixture of Ψ (2.4 mM) and HCHO (53 eq.) in D_2_O at pD 7.5. ^1^H-resonances for 1hmΨ, 3hmΨ, and 1hm3hmΨ are highlighted. (B) ^1^H NMR time course analysis of the same mixture. Formation of the three adducts is completed before the first NMR time point (7 minutes after mixing).

Inosine (I), the N-6 deaminated product of adenosine, arises by spontaneous hydrolysis and by action of deaminase enzymes.^44^ The presence of I in DNA is considered pre-mutagenic and it has functional roles in mRNA and tRNA.^44^ HCHO reacted with I *via* its endocyclic N-1 nitrogen, forming (1-hydroxymethyl)inosine (1hmI); as observed with UMP and TMP, reaction was fast, resulting in establishment of a dynamic equilibrium before the first NMR analysis (Fig. S15†). Ψ, one of most abundant modified nucleosides in RNA,^45^,^46^ also reacted with HCHO *via* either of its endocyclic N-1 and N-3 nitrogens, forming both mono-*N*-hydroxymethylated variants ((1-hydroxymethyl)pseudouridine, 1hmΨ, and (3-hydroxymethyl)pseudouridine 3hmΨ), as well as the dihydroxymethylated adduct (1,3-dihydroxymethyl)pseudouridine (1hm3hmΨ, Fig. 4). Formation of all adducts appeared to reach equilibrium before the first NMR analysis (within 5 min reaction), but their concentrations (0.4 mM for 1hmΨ and 3hmΨ, 0.1 mM for 1hm3hmΨ) were low relative to unreacted Ψ (1.5 mM, Fig. 4B).

### Analyses of the *N*-hydroxymethylated adducts in oligonucleotides

Studies were then carried out on *N*-hydroxymethyl adducts in oligonucleotides. A mixture containing a synthetic Dickerson-Drew-type B-DNA oligonucleotide^47^ (CGCGAATTCGCG, 0.2 mg mL^–1^) and ^13^C-labelled HCHO (0.4 mM) in ammonium formate buffer pH 7.5, and was monitored by ^13^C NMR and 2-dimensional heteronuclear single quantum correlation NMR (2D-^1^H–^13^C-HSQC). No discrete resonances that could be assigned to HCHO-derived adducts with the oligonucleotide were identified; it could be that such species are distributed across different sites, making their detection difficult. However, the intensity of the ^13^C-resonance of hydrated ^13^C-labelled HCHO (*δ*_C_ 82 ppm, Fig. S16†) was significantly lower in the presence of oligonucleotide, implying sequestration of HCHO by the oligonucleotide.

**Fig. 5:**
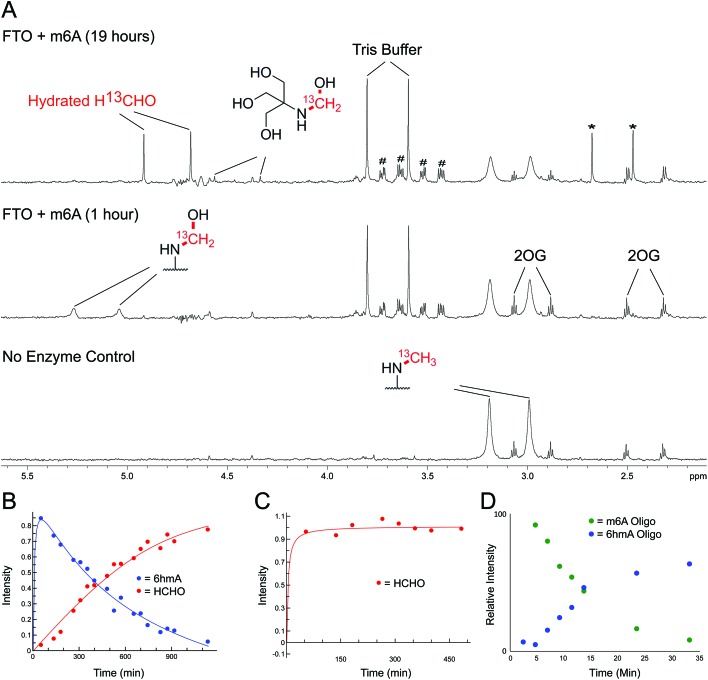
NMR Analyses of FTO-catalysed oxidation of m6A and 3mT. (A) 1-Dimensional ^1^H–^13^C heteronuclear single quantum correlation (1D-^1^H–^13^C-HSQC, ^13^C-coupled) spectra showing FTO-catalysed oxidation of m6A. A sample containing m6A (400 μM), which contained a ^13^C-label on the 6-methyl group, was incubated with 2OG (5 mM), ascorbate (1 mM), ferrous iron (20 μM) and FTO (20 μM) in deuterated ammonium formate buffer in D_2_O at pH 7.5; the reaction was monitored by ^1^H and 1D-^1^H–^13^C-HSQC NMR (over 19 hours, 310 K). Using 1D-^1^H-^13^C-HSQC, ^1^H-resonances from the ^13^C-labelled 6-methyl group are accentuated, enabling sensitive observation of intermediates/products (^1^H-resonances attached to ^13^C-labelled carbon appear as doublets due to ^1^H–^13^C coupling). After one hour, the major ^13^C-labelled product in the reaction was 6hmA; only trace amounts of hydrated ^13^C-labelled HCHO (H^13^CHO) were observed (middle). Formation of 6hmA coincided with decreased m6A and formation of succinate, validating FTO-catalysed hydroxylation of m6A to 6hmA. Over time, 6hmA levels decreased with concomitant H^13^CHO formation (top); no significant increase in succinate was observed after one hour, indicating termination of catalysis. Evidence for formation of ^13^C-labelled methylamine was accrued in the 1D-^1^H–^13^C-HSQC spectrum after 19 hours (asterisks), likely as a consequence of (6-methyl)adenosine deamination; the adduct of residual Tris and H^13^CHO was also observed.^67^ Resonances corresponding to residual HEPES are hashed. (B) 1D-^1^H–^13^C-HSQC time course analysis of FTO-catalysed oxidation of m6A. Levels of 6hmA were highest at the first time point (one hour) before degrading to form H^13^CHO. However, under the tested conditions, 6hmA was more prevalent than H^13^CHO for 6 hours after FTO addition. (C) 1D-^1^H–^13^C-HSQC time course analysis of FTO-catalysed oxidation of 3mT. ^13^C-labelled 3mT (labelled on the 3-methyl group, 400 μM), 2OG (5 mM), ascorbate (1 mM), ferrous iron (20 μM) and FTO (20 μM) were mixed in deuterated ammonium formate buffer in D_2_O at pD 7.5, and the reaction was monitored by ^1^H and 1D-^1^H–^13^C-HSQC NMR over 8 hours at 310 K. Throughout the analysis, the major ^13^C-labelled product was hydrated H^13^CHO; no evidence for the 3hmT species was accrued. (D) MS time course analysis of FTO-catalysed oxidation of m6A-containing RNA oligonucleotide (AUUGUGG-m6A-CUGCAGC). The oligonucleotide (1 μM) was incubated with 2OG (10 μM), ascorbate (100 μM), ferrous iron (10 μM) and FTO (100 nM) in 50 mM Tris buffer in H_2_O at pH 7.5, and the reaction was monitored by MS over 30 min at 298 K. Formation of 6hmA was observed; however, no evidence for the demethylated oligonucleotide was accrued over the time course.

We then analysed the formation and stability of the *N*-hydroxymethylated nucleotide adducts formed *via* nucleic acid demethylase (FTO) catalysis (Fig. 5 and Fig. S17–S20†). FTO was initially reported as a demethylase acting on *N*-methylated DNA,^48^ and subsequently reported to act on *N*-methylated RNA.^49^ FTO catalyses demethylation of (3-methyl)thymine and (6-methyl)adenine in mRNA. Given our observation that 3hmTMP and 6hmdAMP have different formation rates and stabilities, we proposed that the properties of the two FTO-derived products should differ.

FTO-catalysed oxidation of (3-methyl)thymidine (3mT) was first analysed; samples containing 3mT ^13^C-labelled on its 3-methyl group (400 μM), 2OG (5 mM), sodium ascorbate (1 mM), ferrous iron (20 μM) and FTO (20 μM, diluted from a 200 μM stock in HEPES/Tris buffer pH 7.5) were prepared in ammonium formate buffer (D_2_O, pH 7.5), then immediately transferred to a tube for analysis by ^1^H NMR (700 MHz) and 1-dimensional heteronuclear single quantum correlation NMR (1D-^1^H–^13^C-HSQC). 1D-^1^H–^13^C-HSQC analysis allows for enhanced detection of ^1^H resonances attached to ^13^C-labels, and was employed to accentuate resonances derived from the ^13^C methyl group. FTO-catalysed demethylation of 3mT was observed, as evidenced by loss of the *N*-methyl group ^1^H-resonance (doublet at *δ*_H_ 3.3 ppm, *J*_CH_ = 145 Hz), and formation of ^13^C-labelled HCHO (doublet at *δ*_H_ 4.8 ppm, *J*_CH_ = 165 Hz) using 1D-^1^H–^13^C-HSQC (Fig. S17†). No evidence for the presence of 3hmT was accrued, suggesting that either the proposed hydroxymethylated product is unstable (consistent with our HCHO incubation experiments with TMP, Fig. 2 and Fig. S1†), or, less likely, that the demethylation in this case does not proceed *via* initial hydroxylation. Repeating the experiment in the presence of the non-reactive 2OG analogues *N*-oxalylglycine and 2,4-pyridinedicarboxylic acid^38^ inhibited catalysis (90% and 80% inhibition respectively).

Studies with FTO and m6A (^13^C-labelled on its 6-methyl group) under identical conditions over 19 hours also revealed reaction. A competition experiment, with 3mT and m6A both present in the reaction, revealed that 3mT is marginally favoured as a substrate, although similar levels of reactivity with both were observed (Fig. S18†). By contrast with the 3 mT results, the major FTO-catalysed product with m6A during the first 6 hours was clearly the HCHO adduct, *i.e.* (6-hydroxymethyl)adenosine (6hmA); this slowly degraded to adenosine and HCHO (Fig. 5 and Fig. S19†). After the first time point (one hour) no substantial further formation of succinate was observed (using ^1^H NMR, Fig. S20†), suggesting that FTO catalysis was concluded and that formation of HCHO is a consequence of 6hmA fragmentation. Similar results were observed in experiments with an RNA oligonucleotide containing m6A; MS analysis revealed FTO-catalysed hydroxylation of the oligonucleotide, which appeared stable over the course of the experiments (0.5 hours, Fig. 5 and Fig. S21†). This observation is consistent with previous studies on FTO catalysis with m6A in RNA, where MS analyses provided evidence for formation of *N*6-hydroxymethylated and *N*6-formylated adenine in FTO-treated m6A-containing oligonucleotides, as well as in RNA isolated from HeLa cells^50^ (note: we did not observe *N*6-formylation, but this may be due to the different experimental conditions used).

Overall, the NMR and MS observations correlate with the relative stabilities of 3hmTMP and 6hmdAMP in the HCHO incubation experiments, and imply that demethylase catalysis has the potential to result in accumulation of *N*-hydroxymethyl adducts in nucleic acids. Further, the results suggest that, at least *in vitro*, the primary product of FTO catalysis with (6-methyl)adenine is the (6-hydroxymethyl)adenine adduct.

## Conclusions

**Scheme 2:**
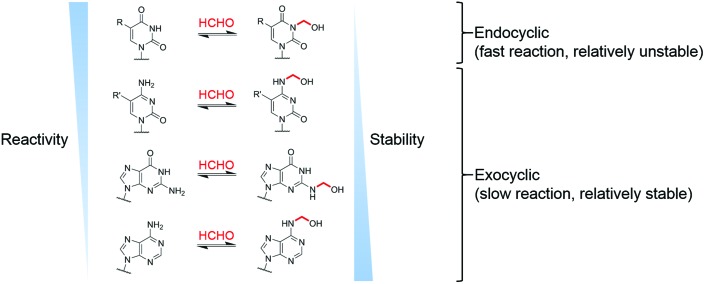
Summary of observed reactions between nucleotides and HCHO. R = H or CH_3_, R′ = H, CH_3_ or CH_2_OH.

Our studies on the reactions of HCHO with nucleobases reveal the formation of hemiaminal adducts *via* reactions on both exocyclic and endocyclic nitrogens, consistent with previous work (Scheme 2).^28^,^29^,^35^,^36^ Incubation of HCHO with TMP and UMP resulted in rapid reaction with the endocyclic nitrogen to form 3-hydroxymethylated derivatives that are in fast dynamic equilibrium with the free nucleotide (Scheme 1 and Fig. 2, Fig. S1 and S2†). Similar reactivity was observed with inosine (I), forming (1-hydroxymethyl)inosine (1hmI, Fig. S15†), and with pseudouridine (Ψ), which strikingly formed three products comprising both 1- and 3-hydroxymethylated adducts, as well as the 1,3-dihydroxymethylated adduct (Fig. 4). The reactive nitrogens in these nucleotides are proposed to predominantly exist as amide N–H groups in their canonical tautomeric forms (as depicted in the figures).^51^ The observed reaction rates for TMP and UMP with HCHO were faster at pD 9 over pD 7.5, consistent with the p*K*_a_ values for cyclic amide N–H bonds (7–10).^52^ The observed fast fragmentation of the hydroxymethyl adducts formed from reactions at the endocyclic nitrogens is also consistent with reported kinetic studies.^53^

By contrast to the reactions of HCHO with TMP, UMP, I and Ψ, those with dCMP, dGMP and dAMP, as well with the modified nucleosides 5mdC, 5hmdC, m6A and 6mG, occur on the exocyclic nitrogens (Scheme 1 and Fig. 2, 3, Fig. S3, S5–S7 and S12–S14†). Whilst slower to form than the adducts with TMP and UMP (correlating with the higher p*K*_a_ values of the exocyclic amines),^52^ these adducts are more stable. In all cases, adduct formation appeared slightly faster at pD 9 and slower at pD 6, relative to pD 7.5 (Fig. S3, S5 and S6†), suggesting the possibility of base catalysis during adduct formation and/or an altering of the dynamicity between the carbonyl and hydrated forms of HCHO. Within limits of detection, no evidence for reactions on endocyclic nitrogens was observed with these nucleotides/nucleosides; this suggests that such reactions either do not occur or that these potential products are unstable. The observations imply that nucleobase hemiaminal adducts are stable enough to have functional roles in biology, potentially relating to their different stabilities and/or rates of formation and breakdown.

While clear reactions between HCHO and nucleic acids to give specific products have been observed, the physiological relevance of such reactions is unknown. Given that HCHO reacts with free nucleotides and with oligonucleotides, and that HCHO is continually produced in cells, it seems probable that intracellular HCHO-DNA/RNA reactions occur, potentially with functional consequences. In addition, it is established that changes in the composition of the cellular nucleotide pool affect DNA/RNA biosynthesis;^54^,^55^ it is thus possible that reactions with HCHO, either on DNA/RNA or free nucleotides, regulate nucleotide reprocessing. The observation of HCHO reactions on endocyclic nitrogens, forming hemiaminal products in fast dynamic equilibrium with the free bases, implies that the hydrogen bonding capabilities of thymine, uracil, inosine and pseudouracil (for duplex formation) could be regulated by HCHO in a concentration-dependent manner. In contrast, reactions on the exocyclic nitrogens of cytosine, adenine and guanine bases do not necessarily block hydrogen bonding interactions (at least in duplex DNA), and therefore, these bases could act as ‘reservoirs’ for HCHO, accumulating HCHO over time.

It is important to consider that within biological contexts the stabilities of the observed adducts may be altered. We accrued no direct evidence for HCHO-derived methylene cross-links, though such species have been proposed in DNA, RNA and between free nucleobases after prolonged incubations;^56^,^57^ we observed low levels of unassigned products. For cross-linking to occur, it is likely that formation of an imine/iminium intermediate is required, which is then ‘trapped’ by a nucleophile, *e.g.* a nucleic acid amine.^27^,^58^,^59^ Such imine/iminium species were not observed in our NMR analyses with nucleotides/nucleosides, implying production of such intermediates might be limiting, at least when HCHO concentrations are low. However, in the case of cellular DNA, where nucleic acid bases are proximal and conformationally constrained, it is possible cross-linking is more efficient, either due to immediate trapping of an imine/iminium ion, or by promoting alternative cross-linking mechanisms. While recent work has shown aminal cross-links between two adenosine molecules are susceptible to rapid hydrolysis, particularly in the presence of anthranilates and phosphanilates, the stability of such species in DNA/RNA is unknown.^57^ It is of interest that histone lysine side chains are proposed to react with (5-formyl)cytosine bases *via* imine formation.^60^–^61^

The effects of the HCHO ‘adducts’ on the cellular functions of nucleobases are yet to be determined, as is how their presence relates to the toxic effects of HCHO. However, the roles of some modifications to nucleobases are well-established, *e.g.* that of (5-methyl)cytosine in the regulation of transcription, and the roles of others are emerging, *e.g.* that of (5-hydroxymethyl)cytosine in transcription and of (6-methyl)adenine in the regulation of RNA stability/activity.^62^,^63^ As well as modulating interactions between nucleic acids, and between nucleic acids and proteins, by formation of covalent cross-links, HCHO adducts have the potential to modulate such interactions by altering non-covalent interactions, *e.g.* by hydrogen-bonding interactions or aqueous solvation. They also have the potential to directly alter the chemical stability of nucleic acids and their oligomers (*e.g.* duplex/quadruplex stability), either directly as shown for (5-methyl)cytosine and (5-hydroxymethyl)cytosine,^47^,^64^ or indirectly by affecting the activity of nucleic acid modifying enzymes such as APOBEC (apolipoprotein B mRNA editing enzyme, catalytic polypeptide-like) and related enzymes.^65^ Whether or not such effects are relevant in cells requires further investigation.

**Scheme 3:**
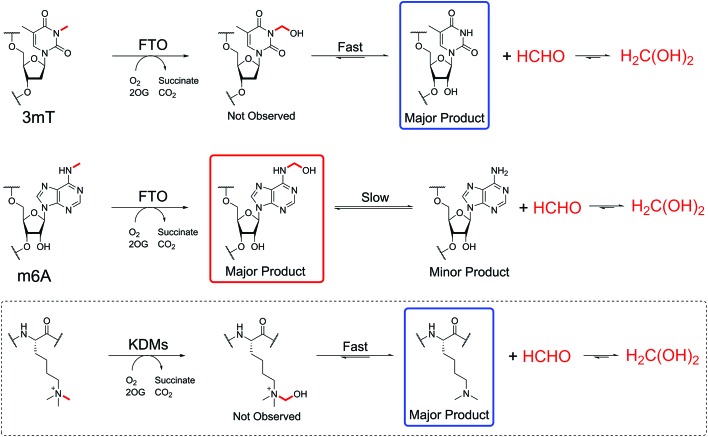
FTO-catalysed oxidation of 3mT and m6A give differently stable *N*-hydroxymethyl products. Treatment of 3mT with FTO leads to formation of 3hmT, which rapidly degrades to the free nucleotide (blue) and HCHO. However, FTO-catalysed hydroxylation of m6A forms 6hmA (red), which only very slowly fragments (over hours). KDM-catalysed demethylation of *N*^*ε*^-methylated lysine residues (dashed box) leads to an unstable hemiaminal that rapidly fragments to the give the demethylated lysine (blue) and formaldehyde (*cf*. 3mT demethylation).

The studies with FTO suggest enzyme-catalysed oxidation of *N*-methylated nucleic acids might be a significant source of *N*-hydroxymethyl adducts in cells, as exemplified by (6-hydroxymethyl)adenine. Oxygenase-catalysed *N*-methyl demethylation has the potential to deliver hemiaminals at specific sites *in vivo*. Further, the results imply that hydroxylation, rather than demethylation, should perhaps be considered as the major biochemical function of FTO catalysis on (6-methyl)adenine (Scheme 3), revealing the possibility that (6-hydroxymethyl)adenine has biologically-relevant functional roles. In this regard, it will be interesting to determine whether oxygenase-catalysed hydroxylation of (6-methyl)adenine may be ‘partnered’ with enzyme-catalysed fragmentation of (6-hydroxymethyl)adenine. It is also possible that the reactions of HCHO with nucleobases are enzyme-catalysed.

Finally, it is likely that the full extent of the reversible reactions between HCHO, and other electrophilic small molecules, with nucleophiles on nucleic acids, proteins, and biological small-molecules is very far from being completely defined. In contrast to the observation of hemiaminal adducts with FTO, adducts have not been observed in studies with *N*^*ε*^-methyllysine demethylases.^39^,^66^ However, work with small molecules reveals the potential for formation of stable HCHO-derived adducts with alkylamines with appropriate stabilisation.^67^ As suggested by the work described here and previously on nucleic acids and some small molecules (*e.g.* glutathione),^68^ there is scope for immense complexity, raising questions about how such interactions may affect and/or regulate the canonical genetic code.

## Conflicts of interest

There are no conflicts to declare.

## Supplementary Material

Supplementary information
