# Supplementary material for: NMR analyses on N-hydroxymethylated nucleobases – implications for formaldehyde toxicity and nucleic acid demethylases
Source: Org Biomol Chem. 2018 May 16;16(21):4021–32. doi: 10.1039/c8ob00734a (PMC5977384; doi:10.1039/c8ob00734a)
Supplement: Supplementary file 1 [file OB-016-C8OB00734A-s001.pdf]

Electronic Supplementary Material (ESI) for Organic & Biomolecular Chemistry.
This journal is © The Royal Society of Chemistry 2018

Supplementary Information

NMR Analyses on *N*-Hydroxymethylated Nucleobases – Implications for Formaldehyde Toxicity and Nucleic Acid Demethylases

Shifali Shishodia[a], Dong Zhang[a], Afaf H. El Sagheer[a], Tom Brown[a], Timothy D. W. Claridge[a], Christopher J. Schofield[a], and Richard J. Hopkinson*[a,b]

[a]*Chemistry Research Laboratory, 12 Mansfield Road, Oxford, OX1 3TA, UK*

[b]*Henry Wellcome Building, Lancaster Road, Leicester, LE1 7RH, UK*

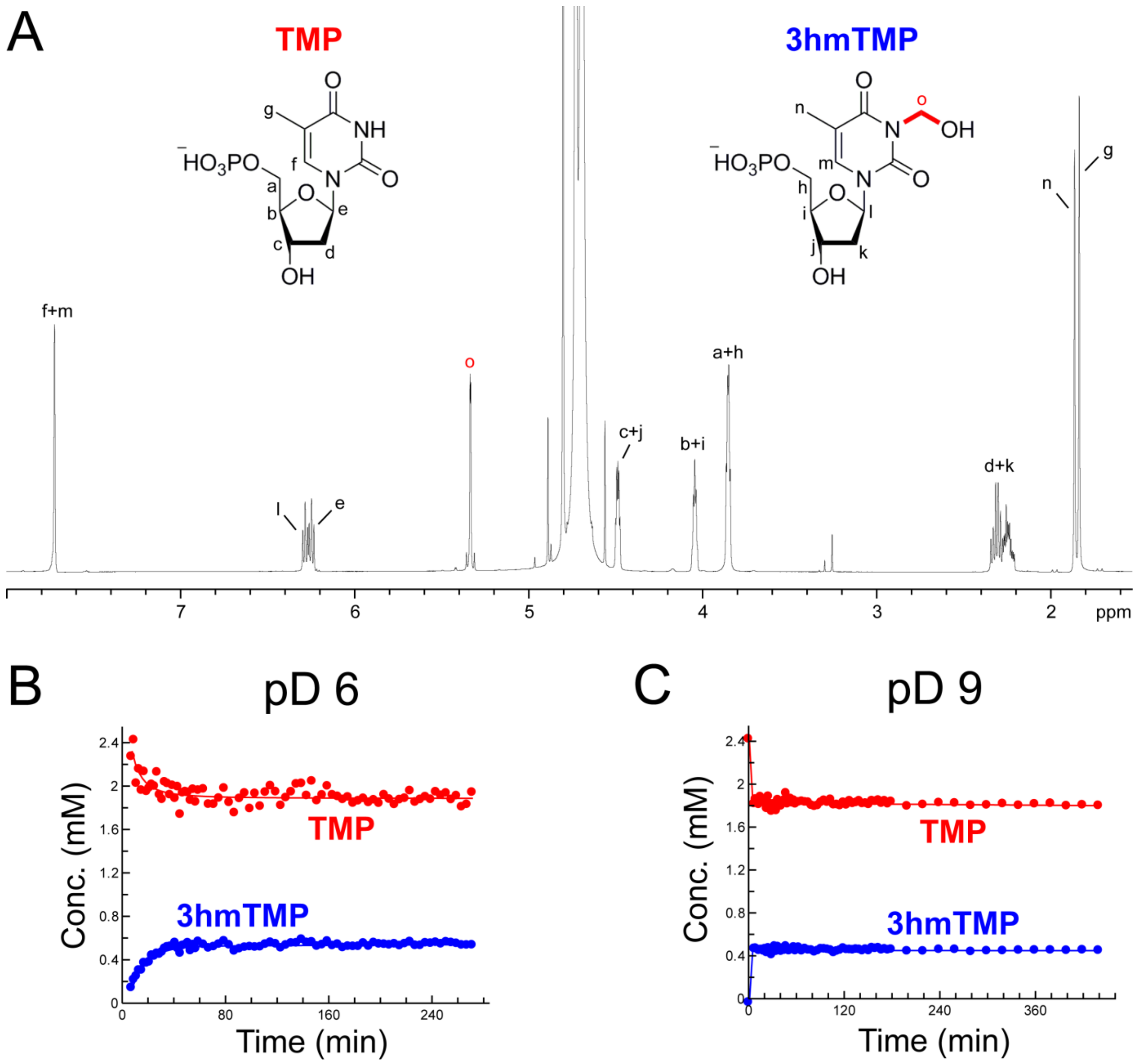

Figure S1. (A) ^{1}H NMR spectrum of a reaction mixture of thymidine monophosphate (TMP) and HCHO in D_2O at pD 7.5. ^{1}H resonances for TMP and (3-hydroxymethyl)thymidine monophosphate (3hmTMP) are highlighted. (B) Graph showing concentrations of TMP and 3hmTMP over time in a reaction mixture of TMP (initial concentration = 2.4 mM) and HCHO (53 equiv.) in D_2O at pD 6. (C) Graph showing concentrations of TMP and 3hmTMP over time in a reaction mixture of TMP (initial concentration = 2.4 mM) and HCHO (53 equiv.) in D_2O at pD 9. The initial rate of 3hmTMP formation is faster at pD 9 than at pD 6 or pD 7.5 (Main Text Figure 2A).

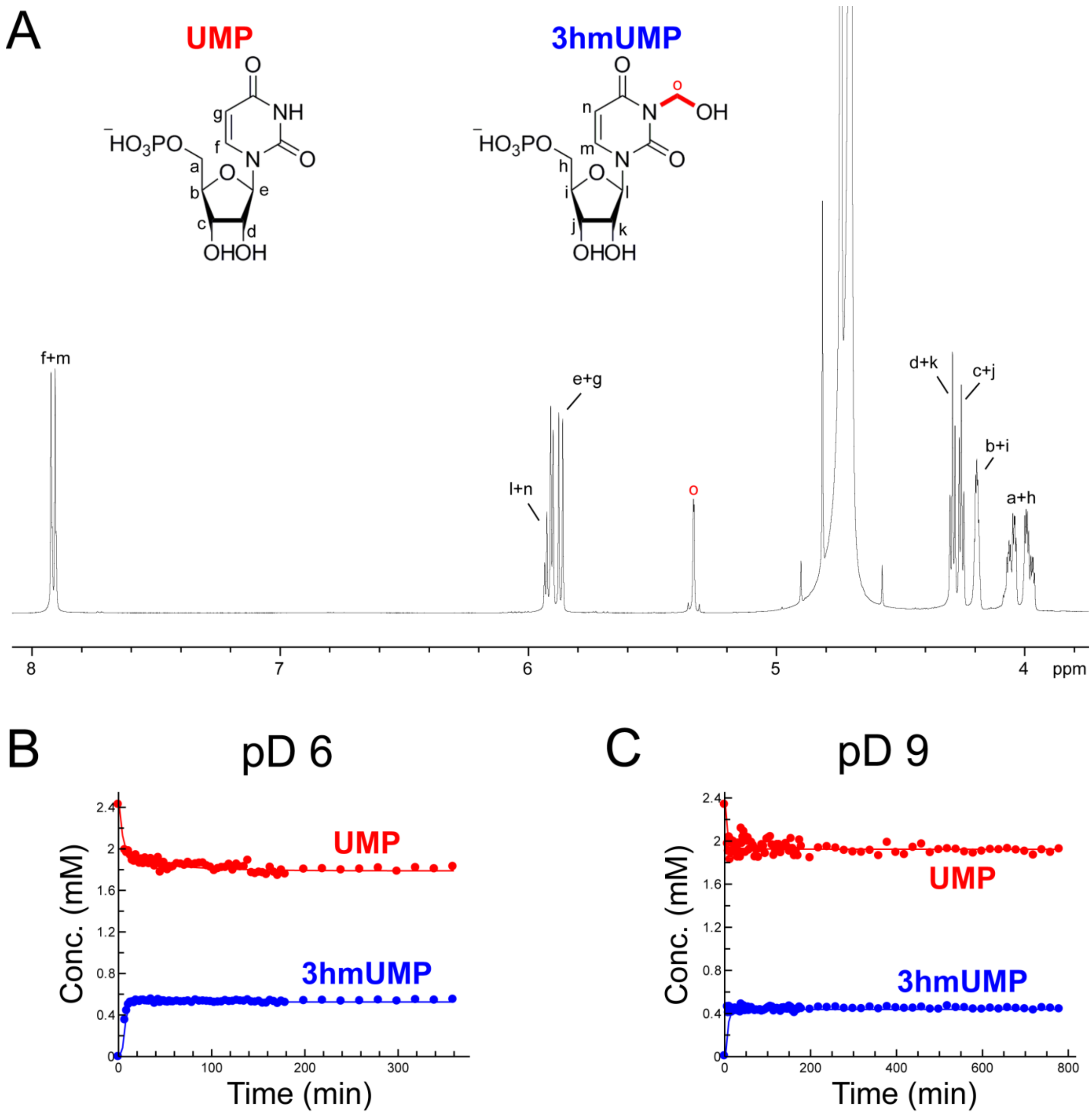

Figure S2. (A) ^{1}H NMR spectrum of a reaction mixture of uridine monophosphate (UMP) and HCHO in D_2O at pD 7.5. ^{1}H resonances for UMP and (3-hydroxymethyl)uridine monophosphate (3hmUMP) are highlighted. (B) Graph showing concentrations of UMP and 3hmUMP over time in a reaction mixture of UMP (initial concentration = 2.4 mM) and HCHO (53 equiv.) in D_2O at pD 6. (C) Graph showing concentrations of UMP and 3hmUMP over time in a reaction mixture of UMP (initial concentration = 2.4 mM) and HCHO (53 equiv.) in D_2O at pD 9. The initial rate of 3hmUMP formation is faster at pD 9 than at pD 6 or pD 7.5 (Main Text Figure 2A).

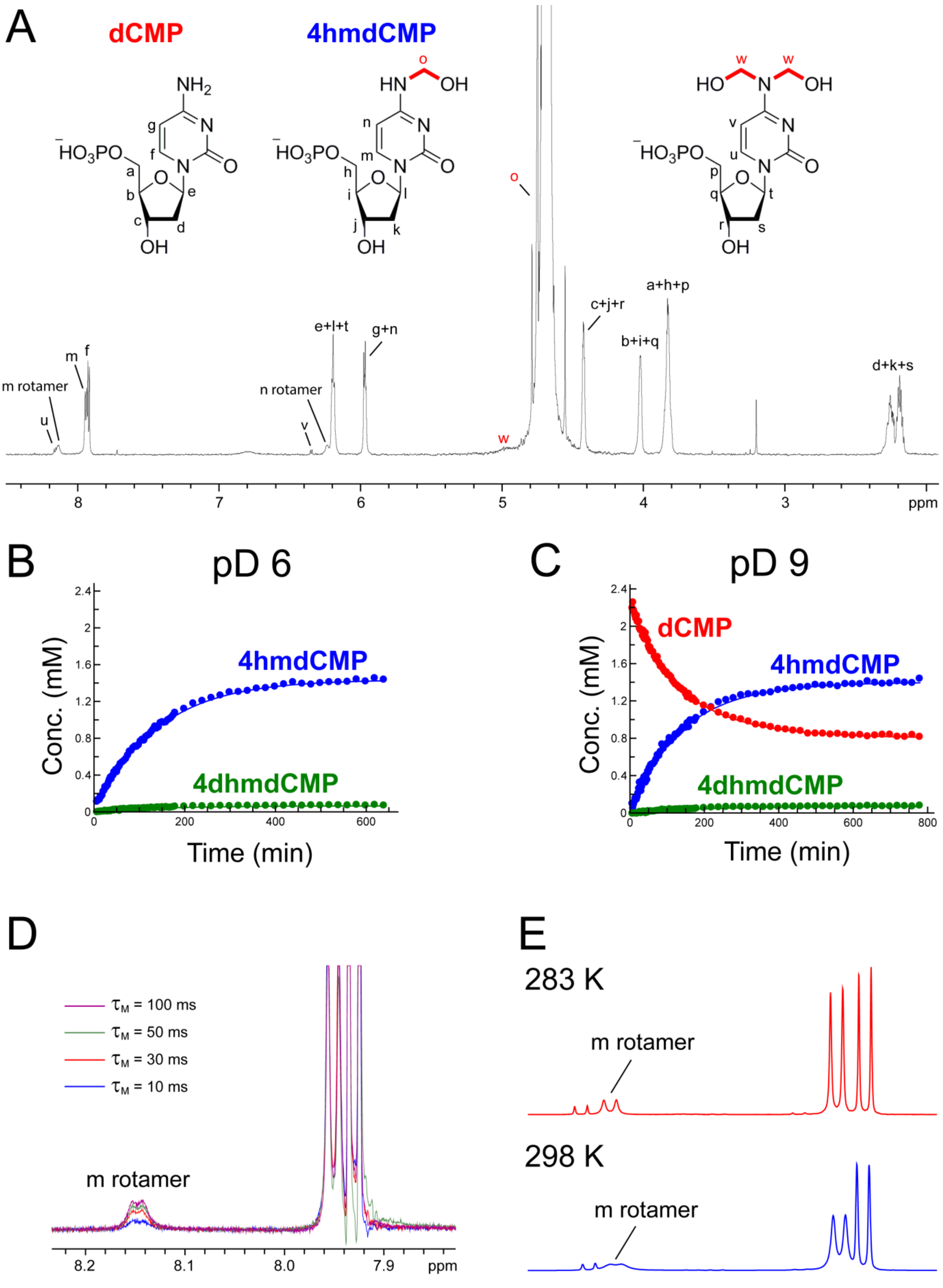

Figure S3. (A) ^{1}H NMR spectrum of a reaction mixture of deoxycytidine monophosphate (dCMP) and HCHO in D_2O at pD 7.5. ^{1}H resonances for dCMP, (4-hydroxymethyl)deoxycytidine monophosphate (4hmdCMP) and (4-dihydroxymethyl)deoxycytidine monophosphate (4dhmdCMP) are highlighted. Evidence for two conformational isomers of 4hmdCMP was observed. (B) Graph showing concentrations of 4hmdCMP and the 4dhmdCMP over time in a reaction mixture of dCMP (initial

concentration = 2.4 mM) and HCHO (53 equiv.) in D_2O at pD 6. The concentration of dCMP could not be accurately determined due to signal overlap. Formation rate of 4hmdCMP = 0.007 mM min^{-1}. Formation rate of 4dhmdCMP = 0.0004 mM min^{-1}. (C) Graph showing concentrations of dCMP, 4hmdCMP and 4dhmdCMP over time in a reaction mixture of dCMP (initial concentration = 2.4 mM) and HCHO (53 equiv.) in D_2O at pD 9. Formation rate of 4hmdCMP = 0.008 mM min^{-1}. Formation rate of 4dhmdCMP = 0.0004 mM min^{-1}. (D) 1-Dimensional EXSY spectra (mixing time (τ_M) = 10-100 ms) of a reaction mixture of deoxycytidine monophosphate (dCMP) and HCHO in D_2O at pD 7.5. Correlations were observed between the resonances at δ_H 7.95 ppm and δ_H 8.15 ppm, which correspond to conformational isomers (rotamers) of 4hmdCMP. (E) ^{1}H NMR spectra of a reaction mixture of deoxycytidine monophosphate (dCMP) and HCHO in D_2O at pD 7.5, at 298 K (bottom) and 283 K (top). Sharpening of the resonances at δ_H 7.95 and δ_H 8.15 ppm was observed upon cooling the mixture, supporting their assignments as conformational isomers.

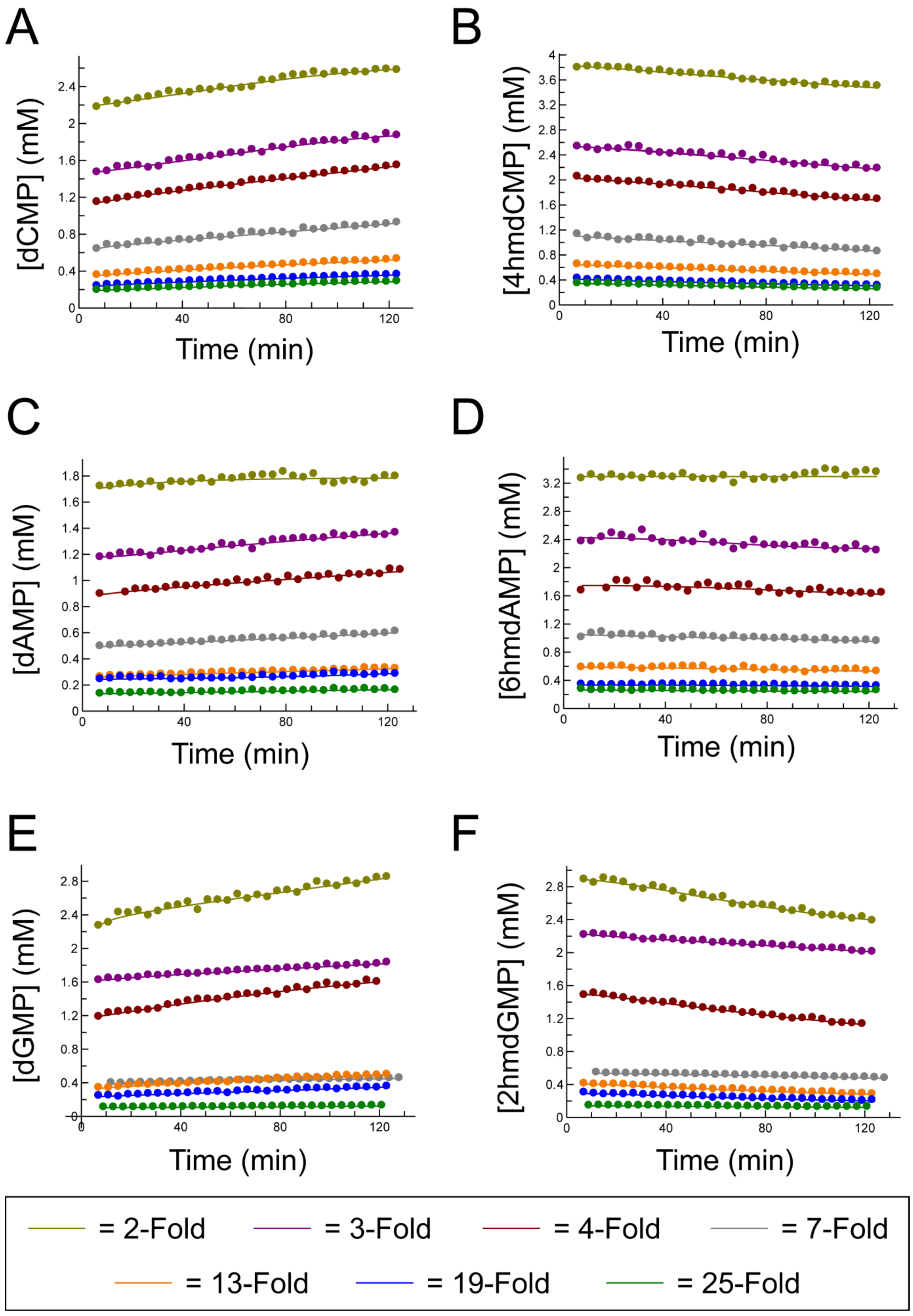

Figure S4. Graphs showing fragmentation rates of monohydroxymethylated HCHO-derived adducts of dCMP (4hmdCMP, B), dAMP (6hmdAMP, D), and dGMP (2hmdGMP, F).

Concomitant formation rates of dCMP (A), dAMP (C) and dGMP (E), after x-fold dilution (as indicated) of reaction mixtures with D_2O (2-fold to 25-fold), are shown.

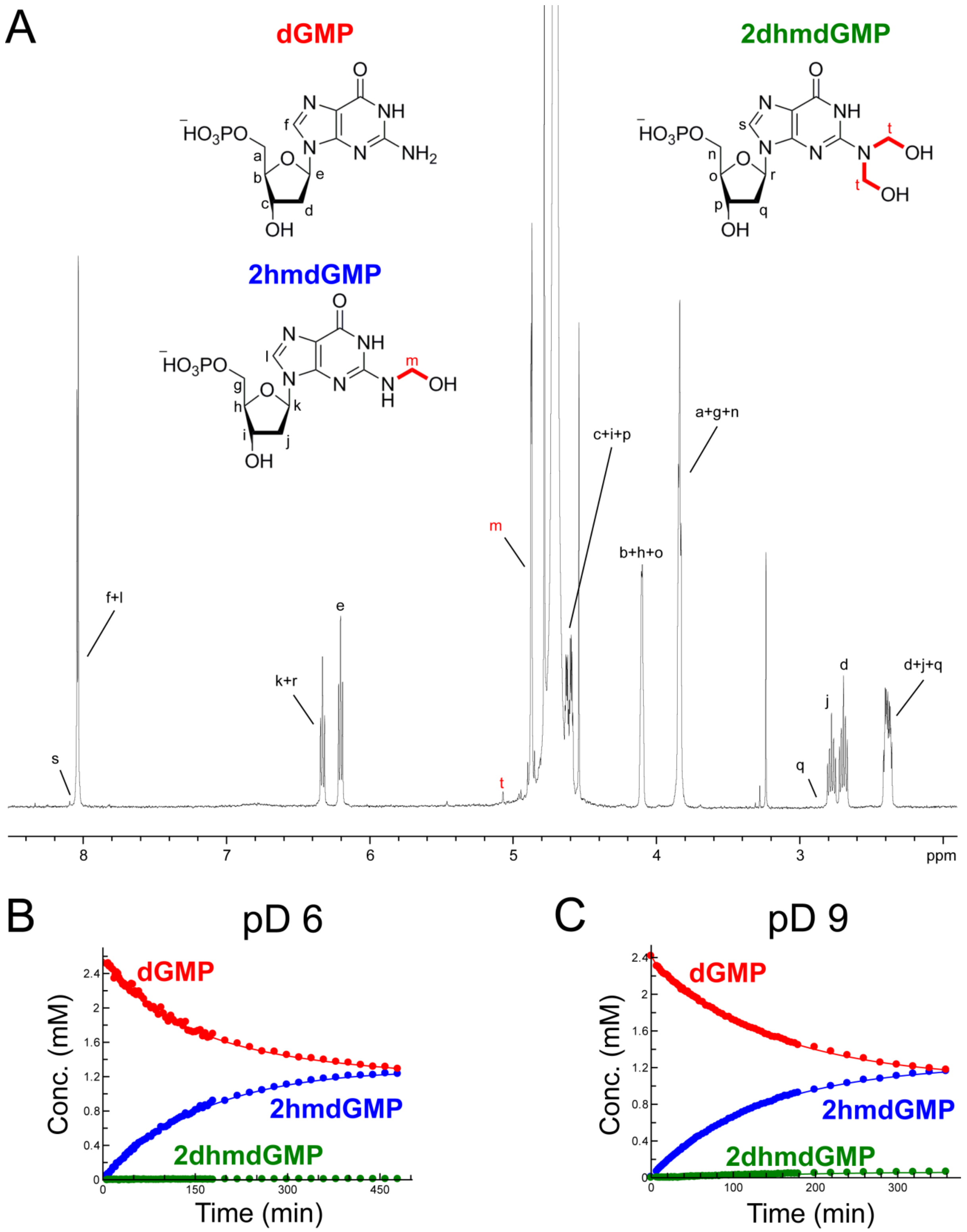

Figure S5. (A) ^{1}H NMR spectrum of a reaction mixture of deoxyguanosine monophosphate (dGMP) and HCHO in D_2O at pD 7.5. ^{1}H resonances for dGMP, (2-hydroxymethyl)deoxyguanosine monophosphate (2hmdGMP) and (2-dihydroxymethyl)deoxyguanosine monophosphate (2dhmdGMP) are highlighted. (B) Graph showing concentrations of dGMP, 2hmdGMP and 2dhmdGMP over time in a reaction mixture of dGMP (initial concentration = 2.4 mM) and HCHO (53 equiv.) in D_2O at pD 6. Formation rate of 2hmdGMP = 0.1 μM s^{-1}. The formation rate of 2dhmdGMP was too low to accurately determine. (C) Graph showing concentrations of dGMP, 2hmdGMP and 2dhmdGMP over time

in a reaction mixture of dGCMP (initial concentration = 2.4 mM) and HCHO (53 equiv.) in D_2O at pD 9. Formation rate of 2hmdGMP = 0.117 µM s^{-1}. Formation rate of 2dhmdGMP = 0.005 µM s^{-1}.

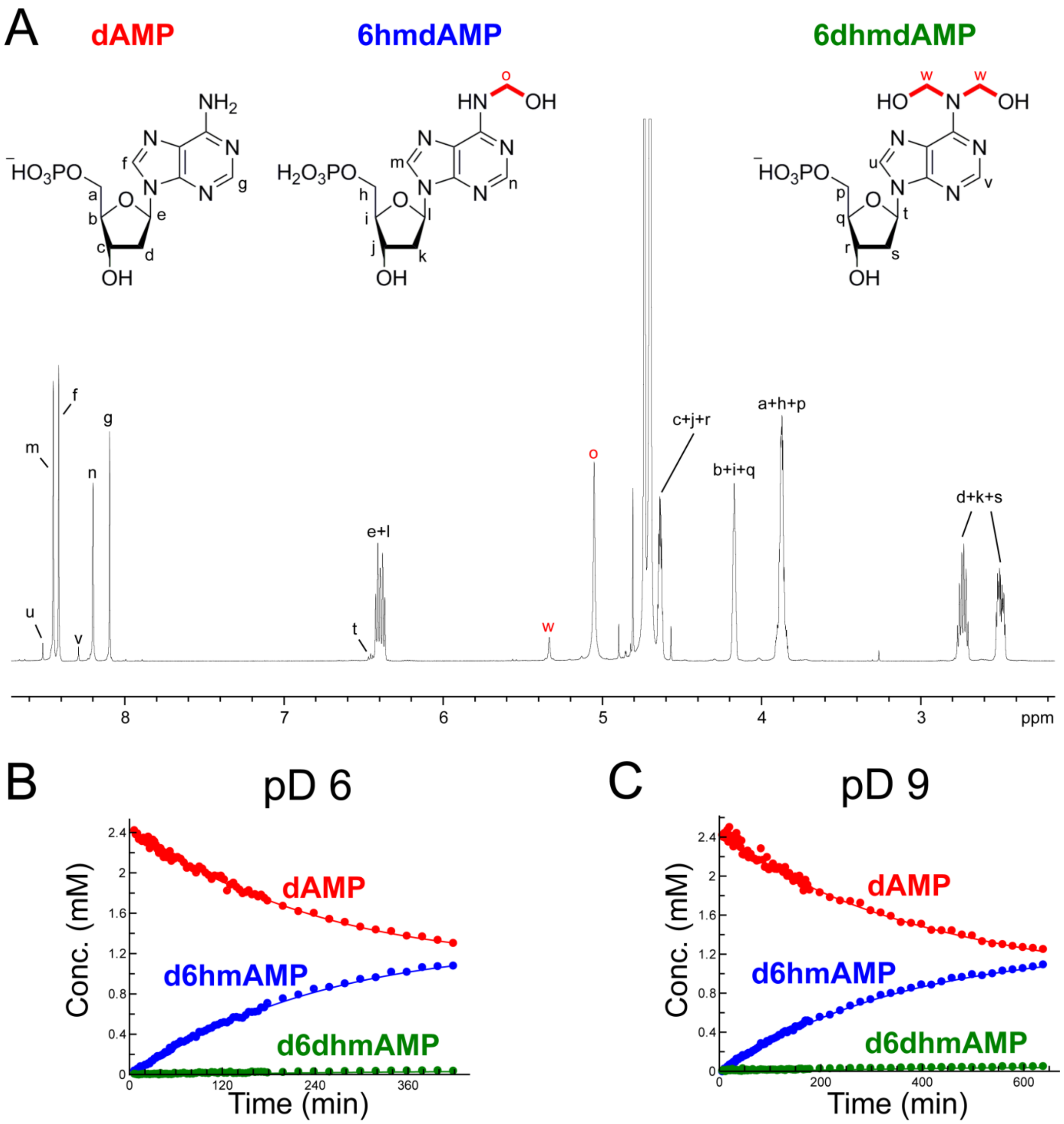

Figure S6. (A) ¹H NMR spectrum of a reaction mixture of deoxyadenosine monophosphate (dAMP) and HCHO in D_2O at pD 7.5. ¹H resonances for dAMP, (6-hydroxymethyl)deoxyadenosine monophosphate (6hmdAMP) and (6-dihydroxymethyl)deoxyadenosine monophosphate (6dhmdAMP) are highlighted. (B) Graph showing concentrations of dAMP, 6hmdAMP and 6dhmdAMP over time in a reaction mixture of dAMP (initial concentration = 2.4 mM) and HCHO (53 equiv.) in D_2O at pD 6. (C) Graph showing concentrations of dAMP, 6hmdAMP and 6dhmdAMP over time in a reaction mixture of dAMP (initial concentration = 2.4 mM) and HCHO (53 equiv.) in D_2O at pD 9. Formation rate of 6hmdAMP = 0.05 µM s^{-1}. The initial rates of adduct formation appear similar at pDs 6, 7.5 and 9.

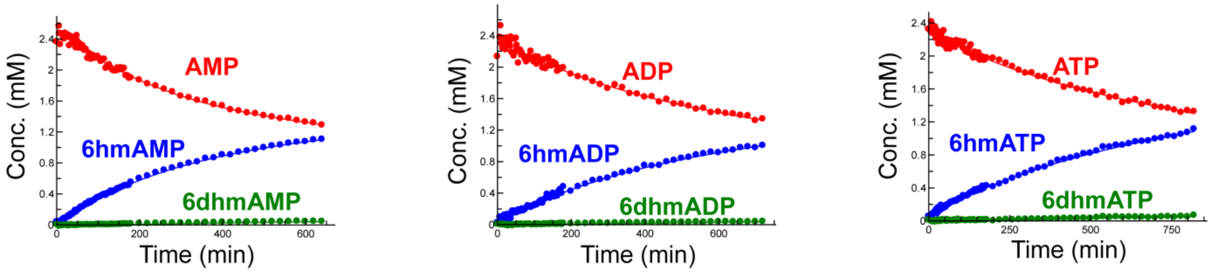

Figure S7. Time courses of reaction mixtures containing adenosine monophosphate (AMP, left), adenosine diphosphate (ADP, middle) and adenosine triphosphate (ATP, right), all at 2.4 mM, with 53 equiv. of HCHO in D_2O at pD 7.5. Formation rate of 6hmAMP = 0.05 μM s^{-1}. Formation rate of 6hmADP = 0.042 μM s^{-1}. Formation rate of 6hmATP = 0.045 μM s^{-1}. The reaction profiles were near-identical to that observed for dAMP and HCHO (see main text Figure 2).

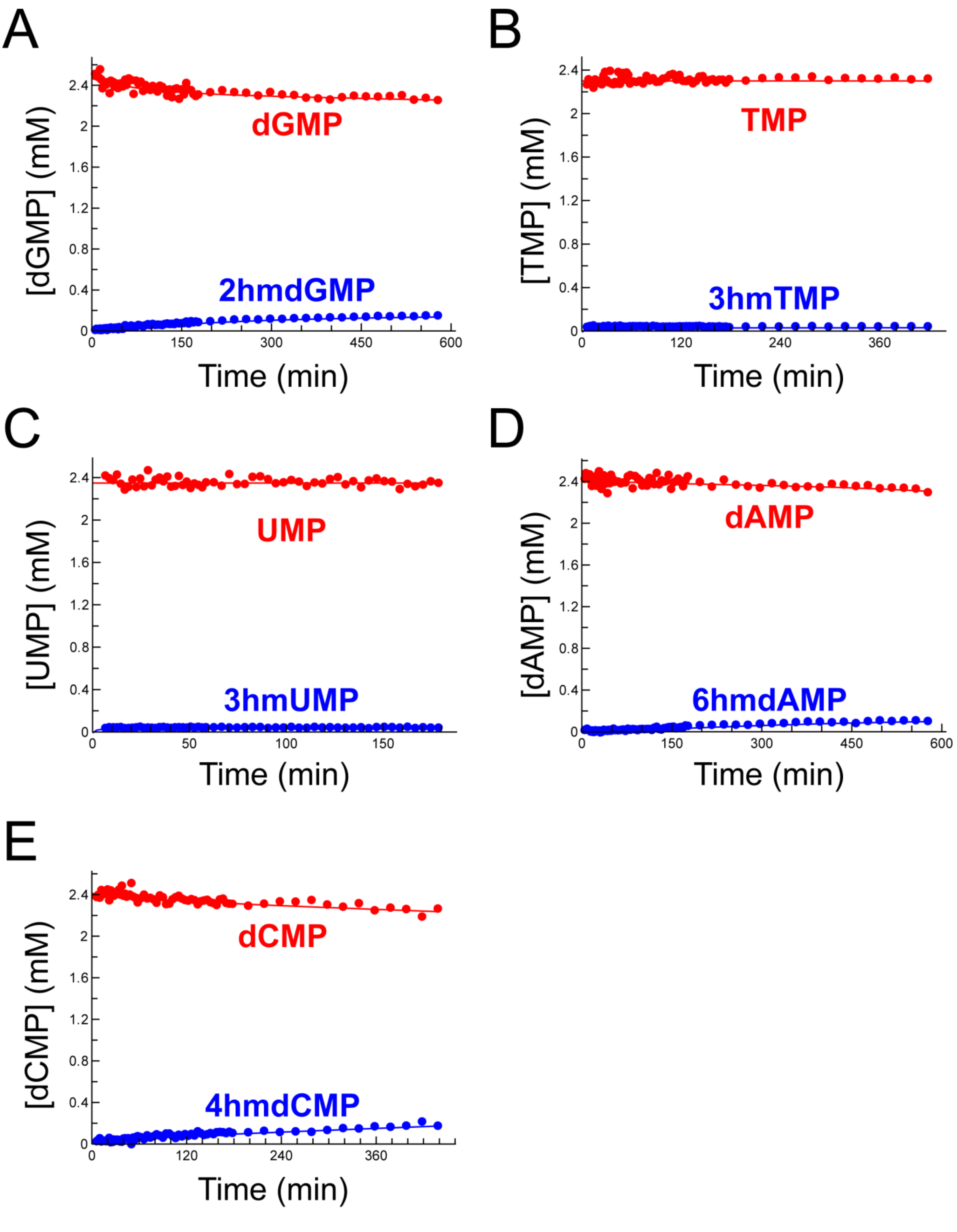

Figure S8. Time courses of reaction mixtures containing canonical nucleotide monophosphates (A: dGMP, B: TMP, C: UMP, D: dAMP, E: dCMP), all at 2.4 mM, with 4 equiv. of HCHO in D_2O at pD 7.5. For mixtures with TMP and UMP (B and C), formation of the respective monohydroxymethylated adducts (3hmTMP and 3hmUMP) was completed before the first NMR analysis, but their equilibrium concentrations were low compared to those observed with the mixtures with 53 equiv. of HCHO (see Main Text Figure 2). Formation of 2hmGMP (A), 6hmdAMP (D) and 4hmdCMP (E) was slower than in mixtures with 53 equiv. of

HCHO (Main Text Figure 2). No evidence for formation of dihydroxymethylated adducts was observed.

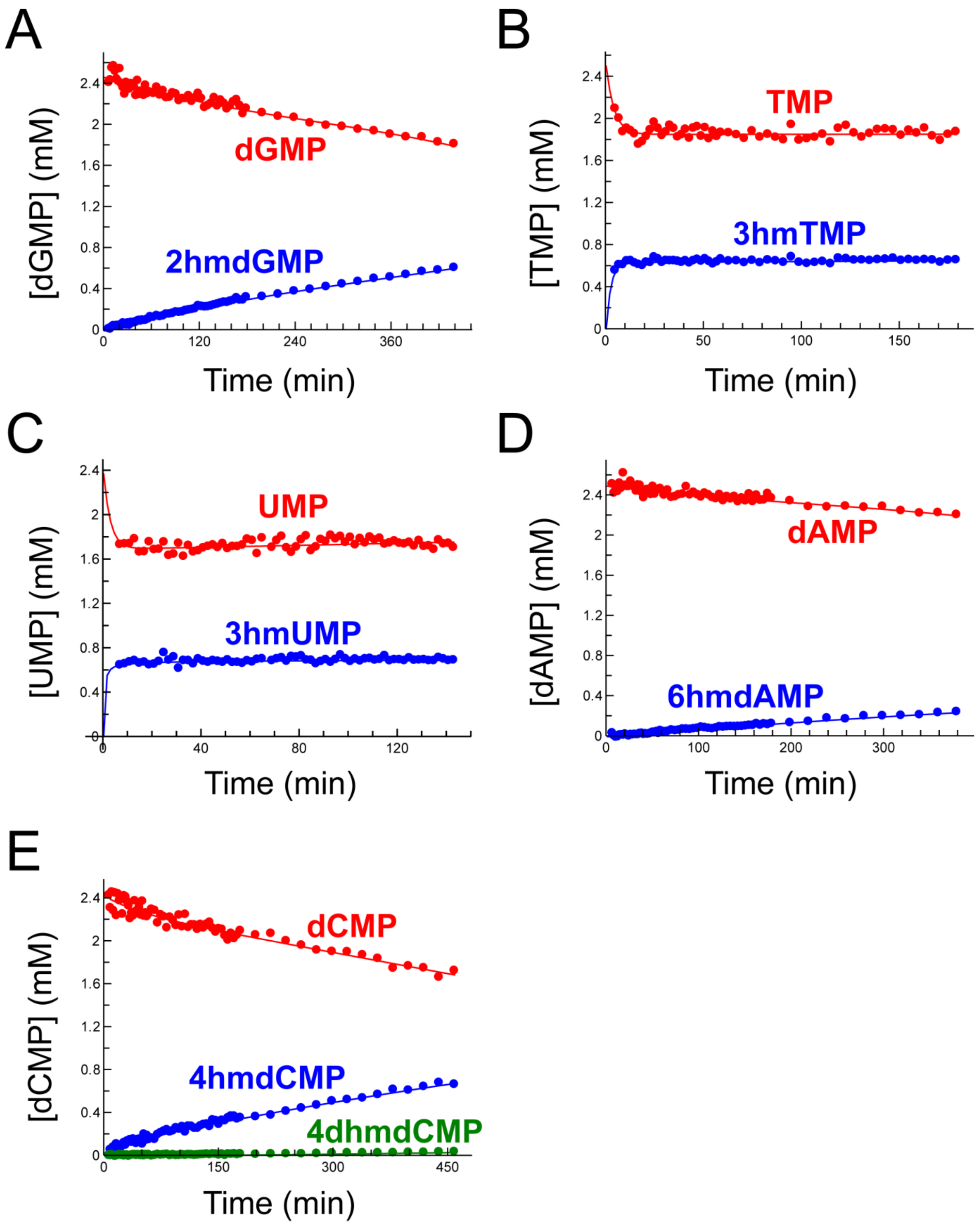

Figure S9. Time courses of reaction mixtures containing canonical nucleotide monophosphates (A: dGMP, B: TMP, C: UMP, D: dAMP, E: dCMP), all at 2.4 mM, with 53 equiv. of HCHO in D_2O at pD 7.5 at 10 °C. Formation of the respective monohydroxymethylated adducts (2hmdGMP, 3hmTMP, 3hmUMP, 6hmdAMP and 4hmdCMP) was slower than in the equivalent reaction mixtures incubated at 25 °C (see Main Text Figure 2). Of the respective dihydroxymethylated adducts, only formation of 4dhmdCMP was observed (at low levels).

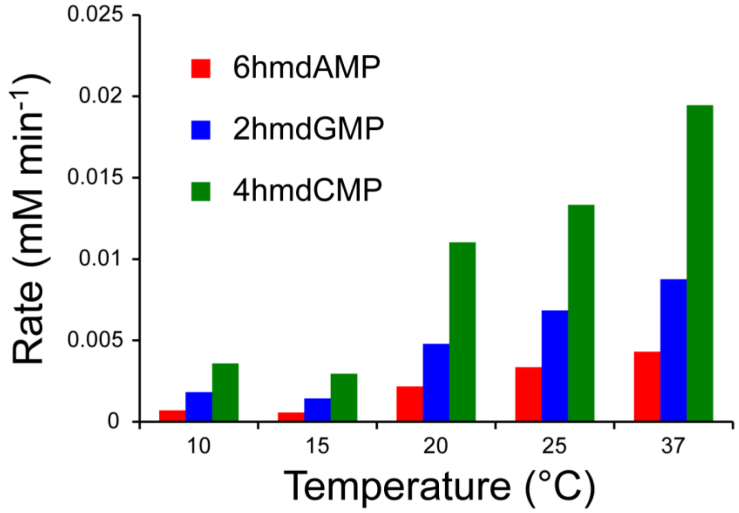

Figure S10. Bar graph showing initial formation rates of 6hmdAMP (red), 2hmdGMP (blue) and 4hmdCMP (green) at different temperatures, from mixtures of HCHO (53 equiv.) with dAMP, dGMP and dCMP respectively (all 2.4 mM). Formation rates for the three adducts show a near-linear temperature dependence (note: determination of accurate formation rates at 10 and 15 °C is difficult due to the low concentrations of the adducts). At all temperatures, formation of 6hmdAMP is slowest (0.067 µM s^{-1} at 37 °C), followed by 2hmdGMP (0.15 µM s^{-1} at 37 °C) and 4hmdCMP (0.317 µM s^{-1} at 37 °C).

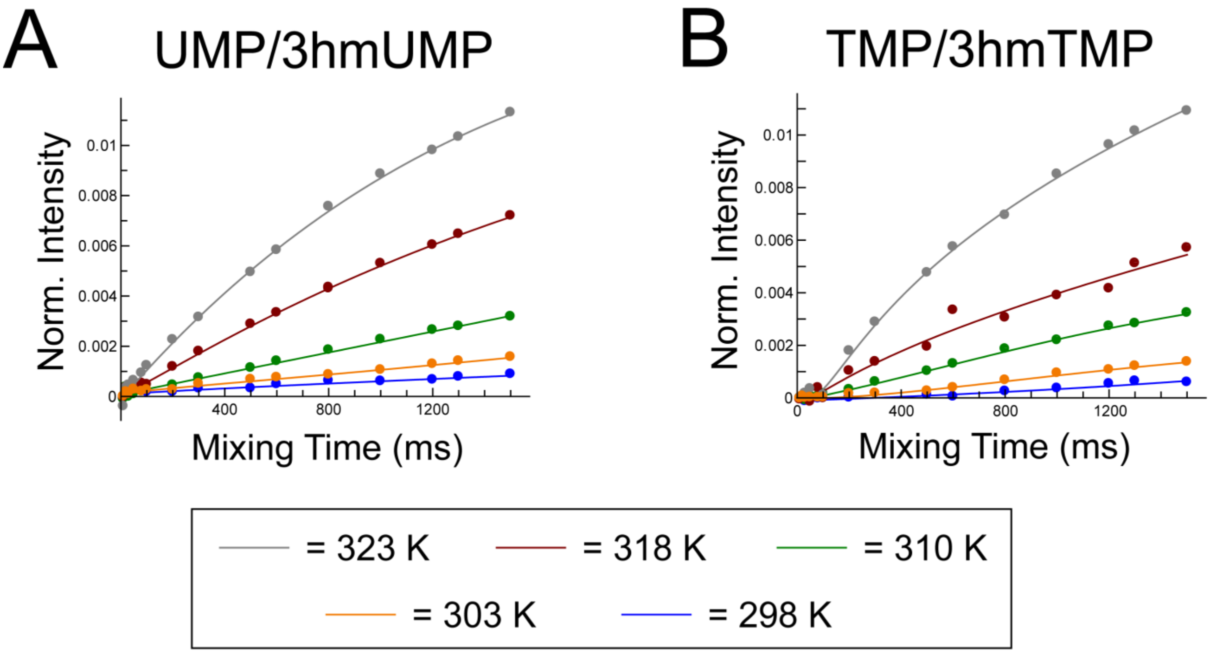

C

	UMP		TMP	
Temperature (K)	k_1 (mM^{-1} min^{-1})	k_{-1} (min^{-1})	k_1 (mM^{-1} min^{-1})	k_{-1} (min^{-1})
298	18716	8143	12648	5548
303	32191	15255	16833	10255
310	69240	36326	64054	49489
318	157375	121998	135127	80907
323	285662	184078	299398	194600

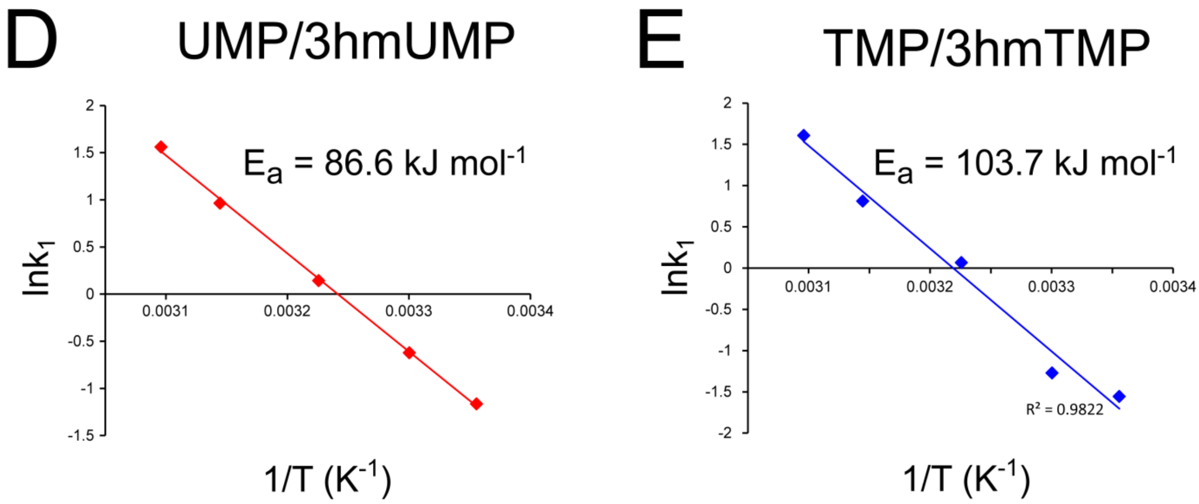

Figure S11. (A and B) Graphs showing EXSY correlation intensities in reaction mixtures of UMP and TMP with HCHO, recorded at different mixing times (τm). Build-up rates correlate with adduct formation rates at equilibrium. (C) Table showing calculated rate constants for 3hmUMP/3hmTMP formation (k_1) and hydrolysis (k_{-1}) at different temperatures. (D and E) Plots of lnk_1 versus 1/temperature for UMP/3hmUMP and TMP/3hmTMP. Calculated activation energies (E_a) are shown on the plots.

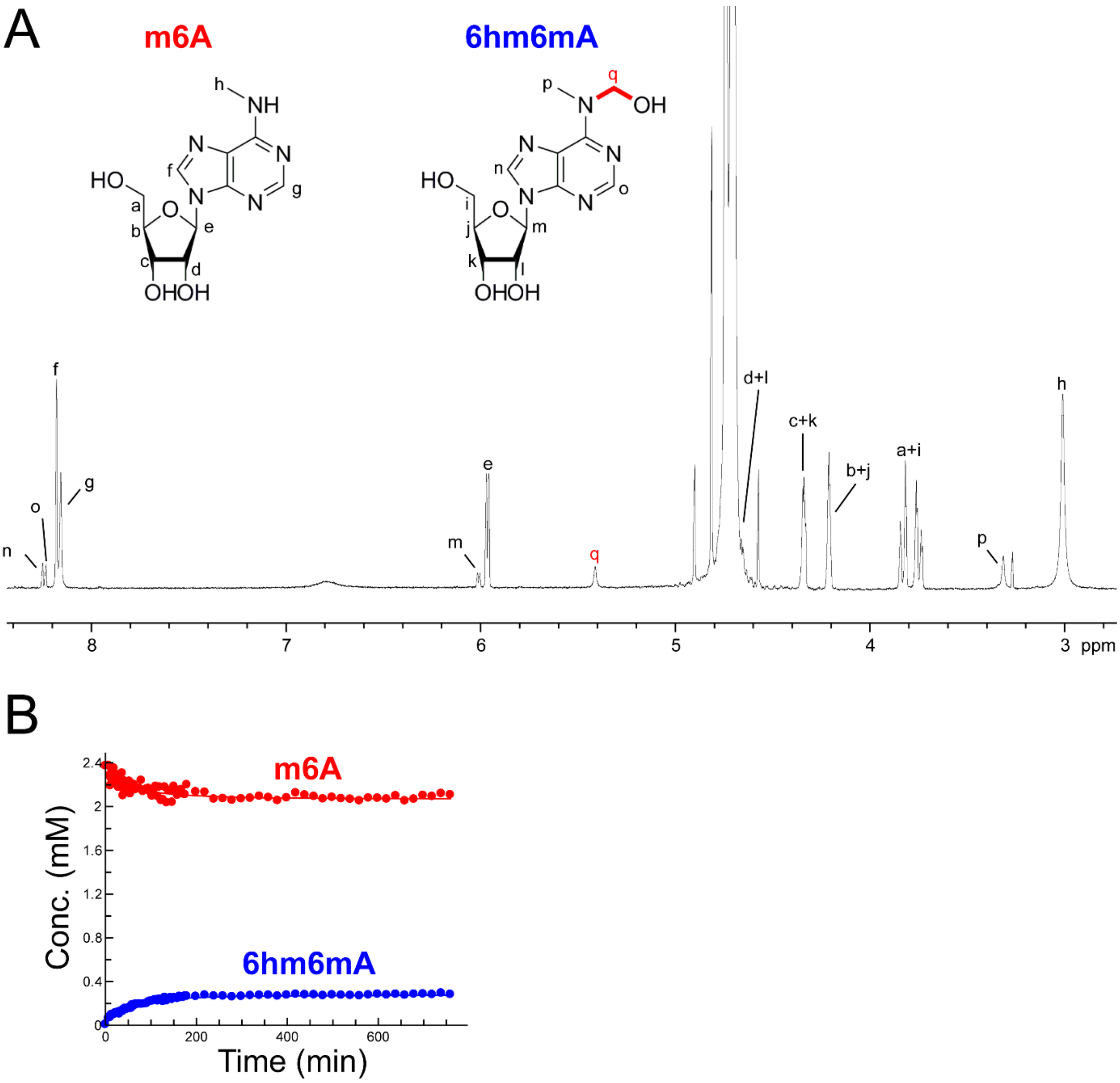

Figure S12. (A) ^{1}H NMR spectrum of a reaction mixture of (6-methyl)adenosine (m6A) and HCHO in D_2O at pD 7.5. ^{1}H resonances for m6A and (6-hydroxymethyl-6-methyl)adenosine (6hm6mA) are highlighted. (B) Graph showing concentrations of m6A and 6hm6mA over time for reaction of m6A (initial concentration = 2.4 mM) and HCHO (53 equiv.) in D_2O at pD 7.5.

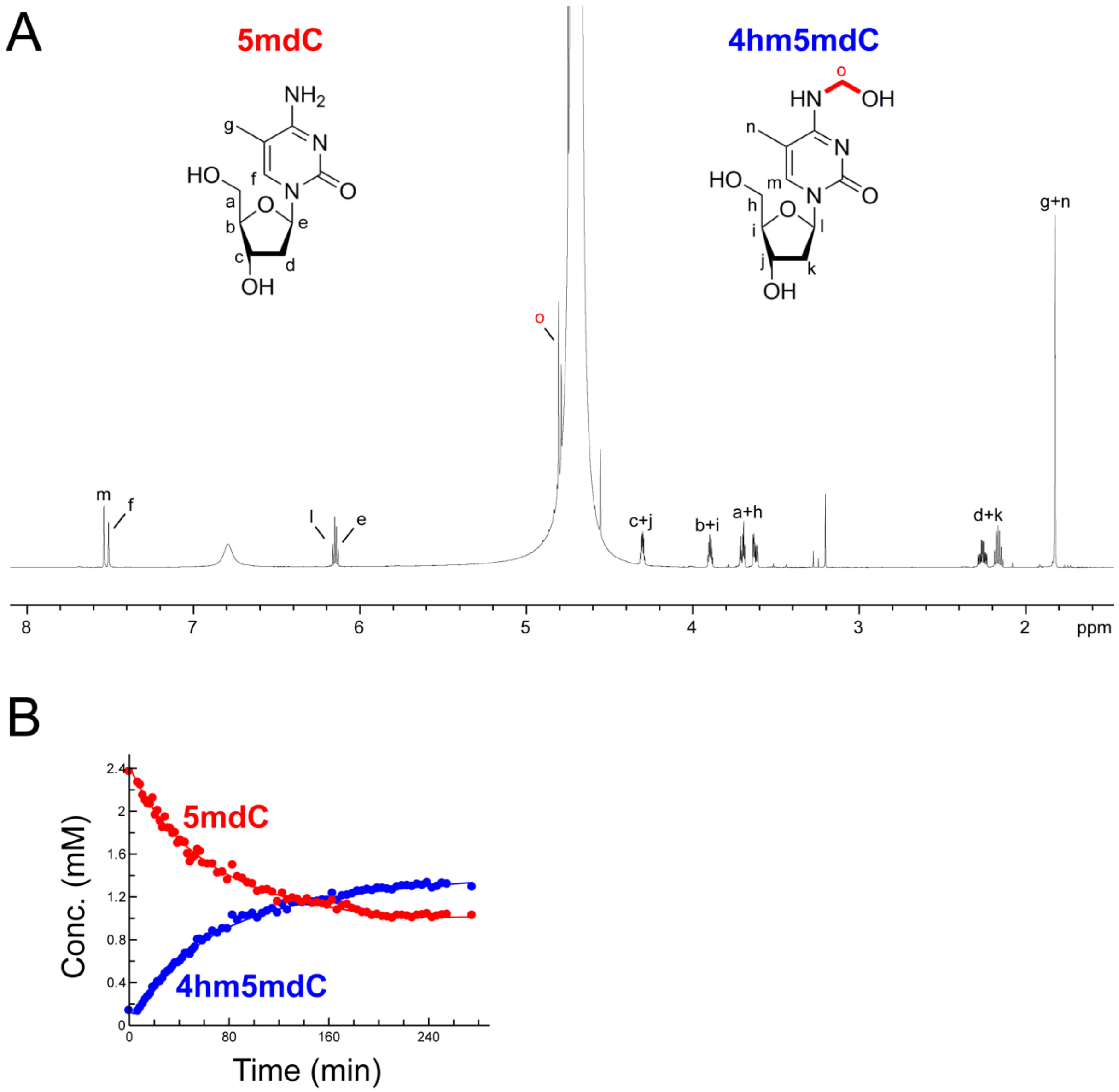

Figure S13. (A) ^{1}H NMR spectrum of a reaction mixture of (5-methyl)deoxycytidine (5mdC) and HCHO in D_2O at pD 7.5. ^{1}H resonances for 5mdC and (4-hydroxymethyl-5-methyl)deoxycytidine (4hm5mdC) are highlighted. (B) Graph showing concentrations of 5mdC and 4hm5mdC over time for reaction of 5mdC (initial concentration = 2.4 mM) and HCHO (53 equiv.) in D_2O at pD 7.5.

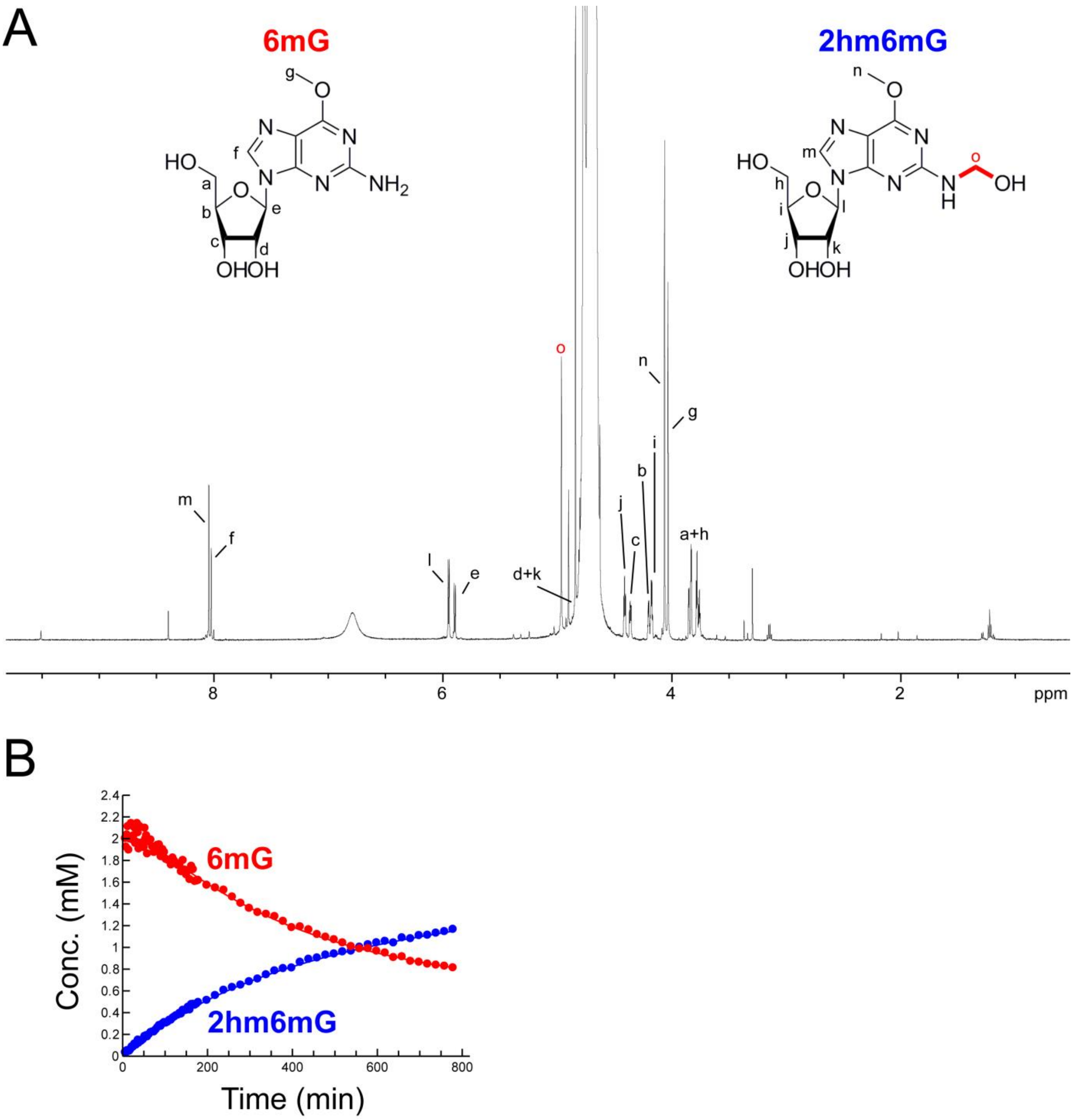

Figure S14. (A) ^{1}H NMR spectrum of a reaction mixture of (6-methyl)guanosine (6mG) and HCHO in D_2O at pD 7.5. ^{1}H resonances for 6mG and (2-hydroxymethyl-6-methyl)guanosine (2hm6mG) are highlighted. (B) Graph showing concentrations of 6mG and 2hm6mG over time in a reaction mixture of 6mG (initial concentration = 2.4 mM) and HCHO (53 equiv.) in D_2O at pD 7.5.

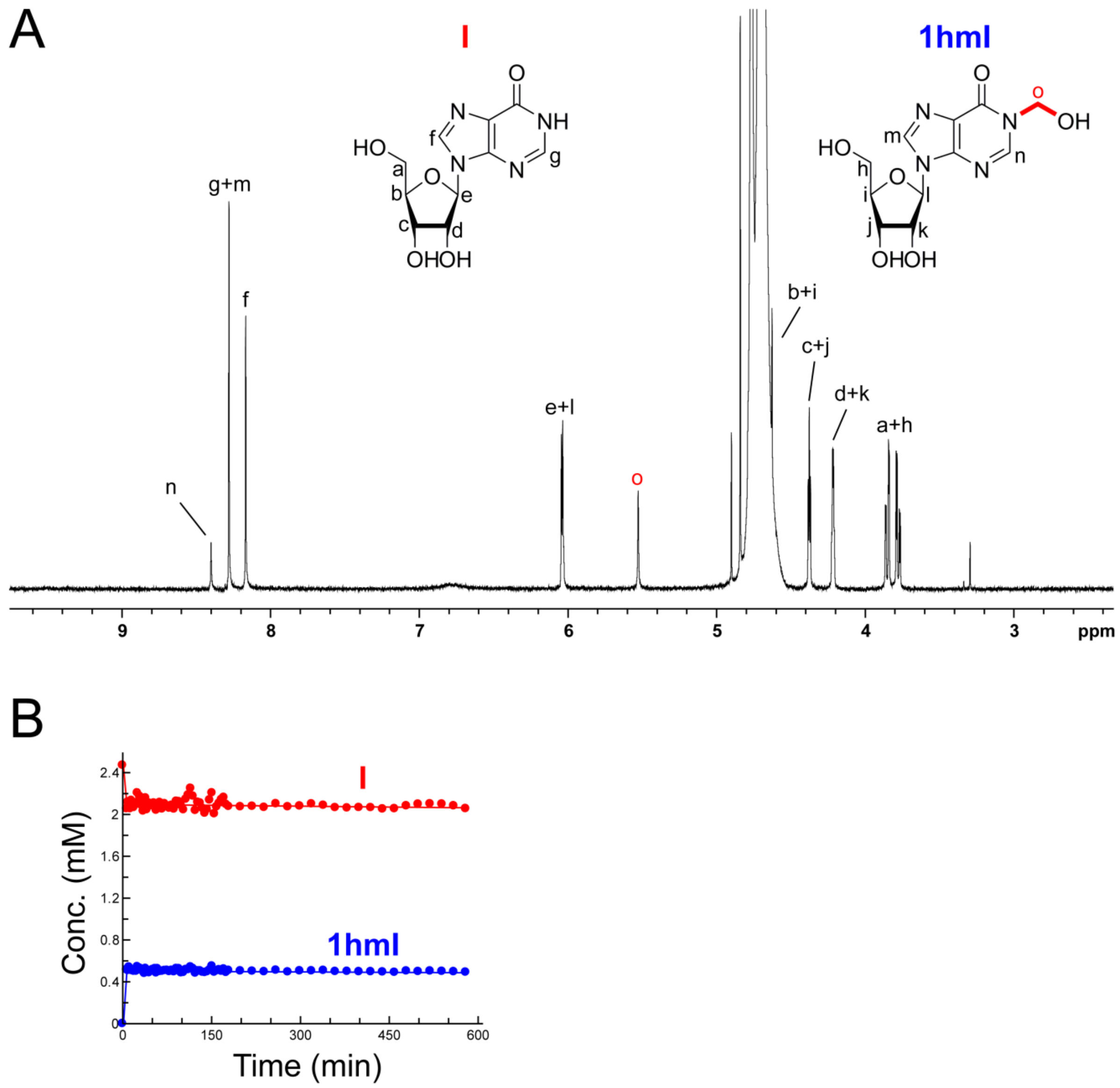

Figure S15. (A) ^{1}H NMR spectrum of a reaction mixture of inosine (I) and HCHO in D_2O at pD 7.5. ^{1}H resonances for I and (1-hydroxymethyl)inosine (1hmI) are highlighted. (B) Graph showing concentrations of I and 1hmI over time in a reaction mixture of I (initial concentration = 2.4 mM) and HCHO (53 equiv.) in D_2O at pD 7.5.

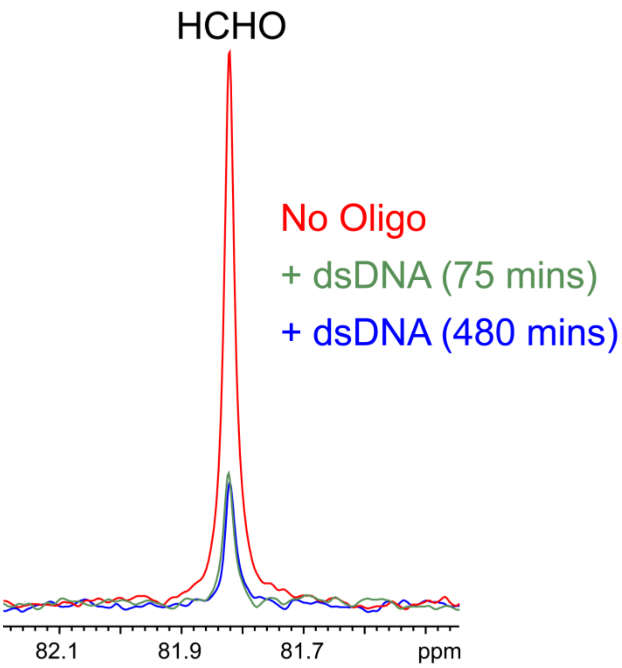

Figure S16. Overlay of ^{13}C NMR spectra of ^{13}C-labelled HCHO ($H^{13}CHO$) in 19.5 mM ammonium formate buffer pH 7.5 (+ 10 % v/v D_2O), in the presence of either H_2O (red, after 12 hours) or 0.2 mg/mL double-stranded DNA oligomer in H_2O (dsDNA, sequence CGCGAATTCGCG; green, after 75 mins; blue, after 8 hours)). The loss of signal intensity implies sequestration of $H^{13}CHO$ by the DNA.

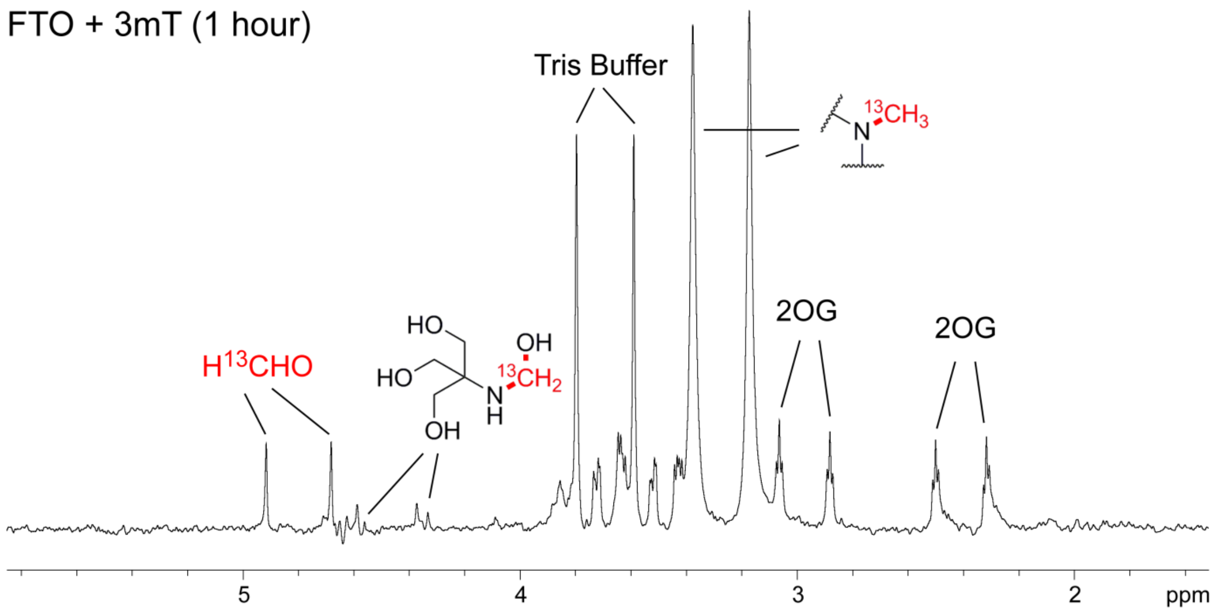

Figure S17. 1-Dimensional ^{1}H-^{13}C heteronuclear single quantum correlation (1D-^{1}H-^{13}C-HSQC, ^{13}C-coupled) spectrum showing FTO-catalysed oxidation of (3-methyl)thymidine (3mT), ^{13}C-labelled on the 3-methyl group, after 1 hour. Resonances corresponding to ^{13}C-labelled HCHO ($H^{13}CHO$), the methyl group of 3mT, Tris buffer, a Tris-HCHO adduct and 2-oxoglutarate (2OG) are highlighted. No evidence for (3-hydroxymethyl)thymidine was accrued; by contrast studies with (6-methyl)adenosine and FTO gave (6-hydroxymethyl)adenosine as the major product. For details on sample preparation, see below.

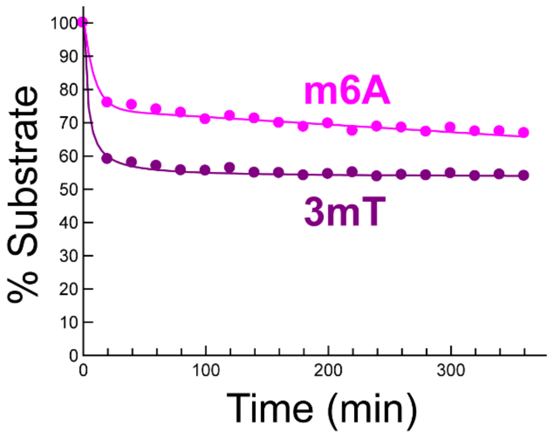

Figure S18. Time-course graph showing reduction in ^{13}C-labelled (3-methyl)thymidine (3mT) and (6-methyl)adenosine (m6A) upon incubation with FTO (as determined by 1D-^{1}H-^{13}C-HSQC analysis). This preliminary data suggests 3mT is a marginally better substrate than m6A.

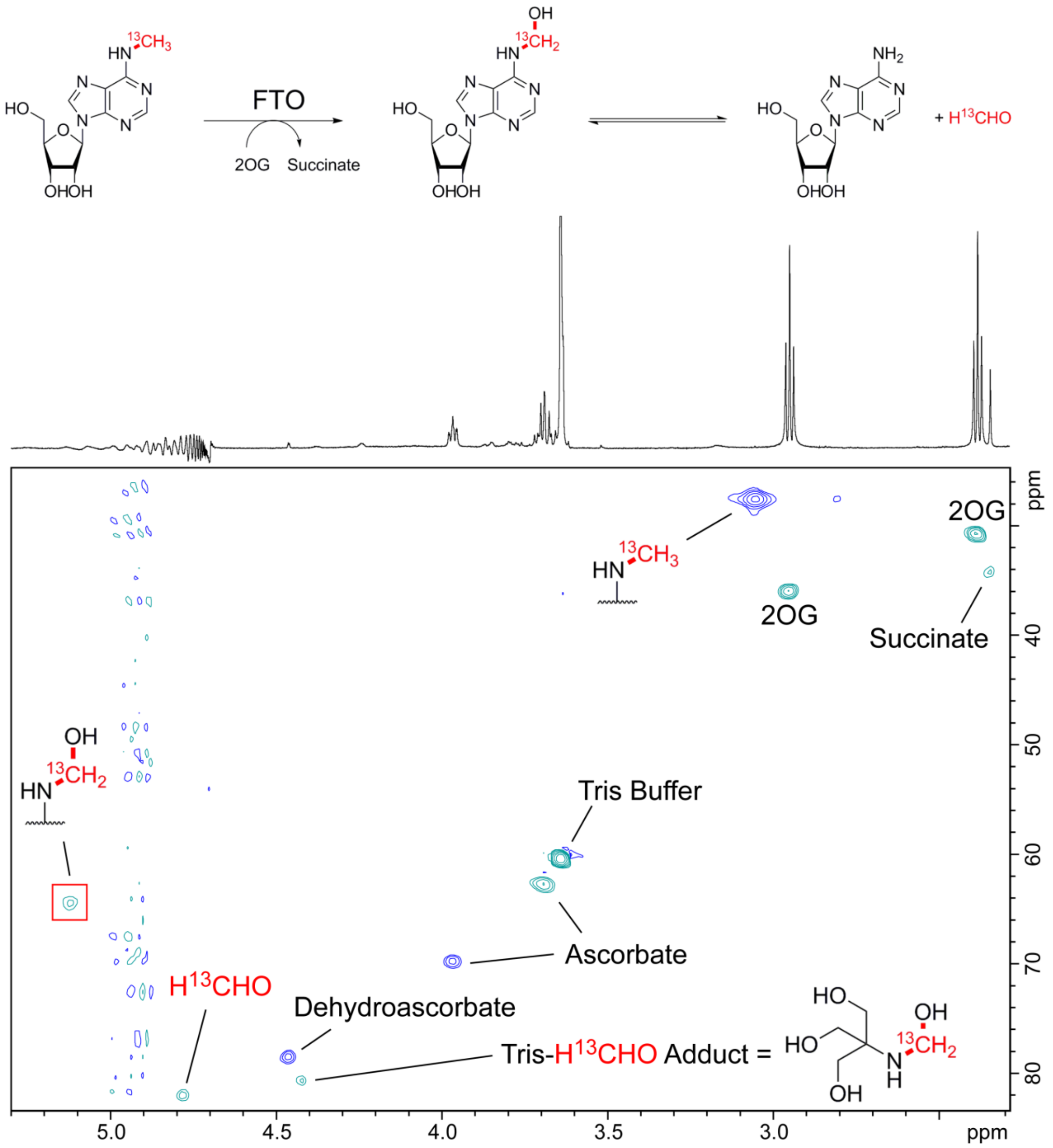

Figure S19. 2-Dimensional ^{1}H-^{13}C heteronuclear single quantum coherence (2D-^{1}H-^{13}C-HSQC, ^{13}C-decoupled) spectrum showing FTO-catalysed oxidation of (6-methyl)adenosine (m6A), ^{13}C-labelled on the 6-methyl group. Resonances corresponding to hydrated ^{13}C-labelled HCHO ($H^{13}CHO$), the hemiaminal CH_2 of (6-hydroxymethyl)adenosine (6hmA, red box), the methyl group of m6A, Tris buffer, a Tris-HCHO adduct, 2-oxoglutarate (2OG), succinate, ascorbate and dehydroascorbate are highlighted. Green resonances correspond to CH_2 groups, while blue resonances correspond to either CH or CH_3 groups.

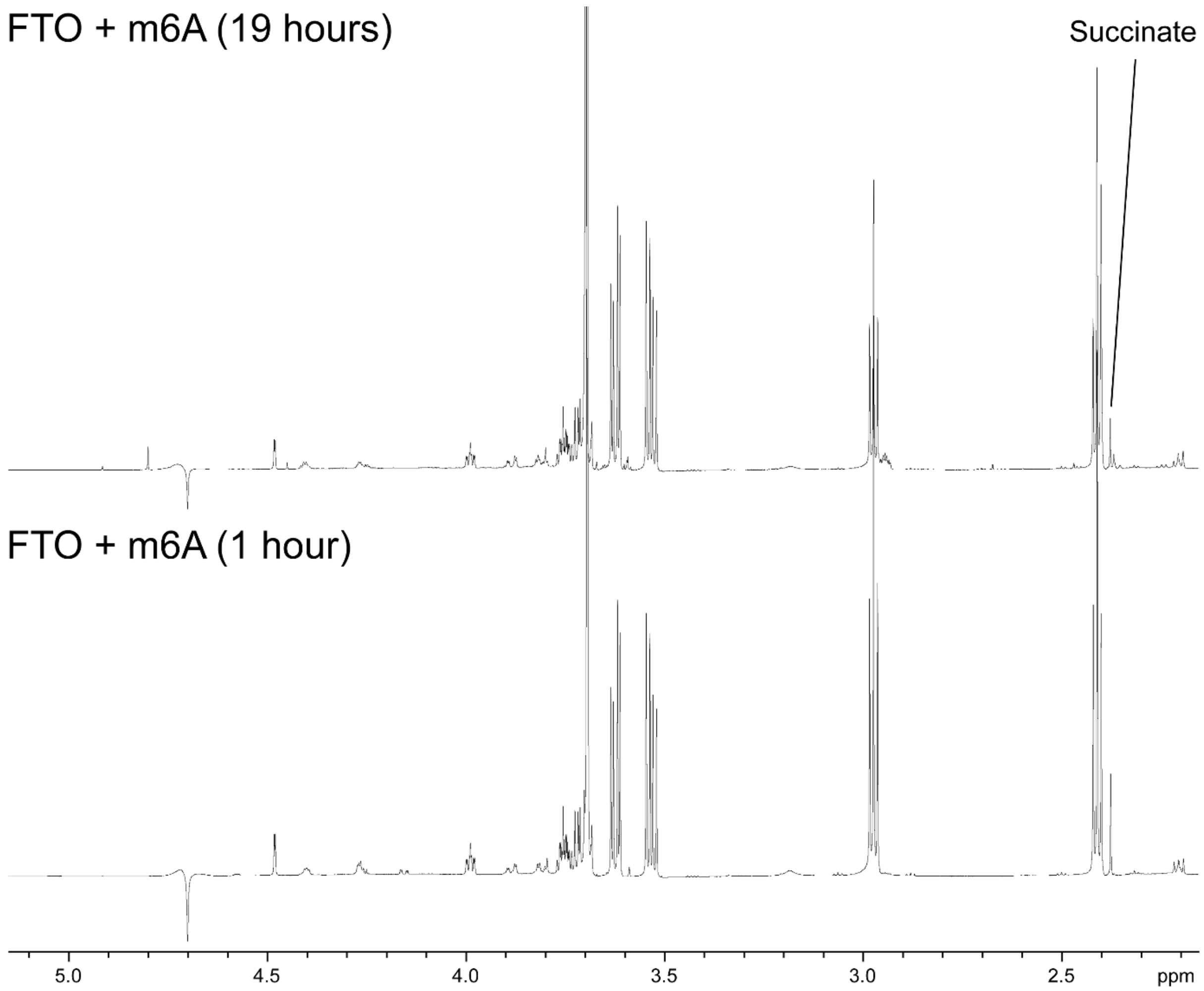

Figure S20. ^{1}H NMR spectrum showing FTO-catalysed oxidation of (6-methyl)adenosine (m6A), ^{13}C-labelled on the 6-methyl group, after 1 hour and 19 hours (1D-^{1}H-^{13}C-HSQC spectra are shown in Figure 5 of the Main Text). The ^{1}H resonance corresponding to succinate is highlighted.

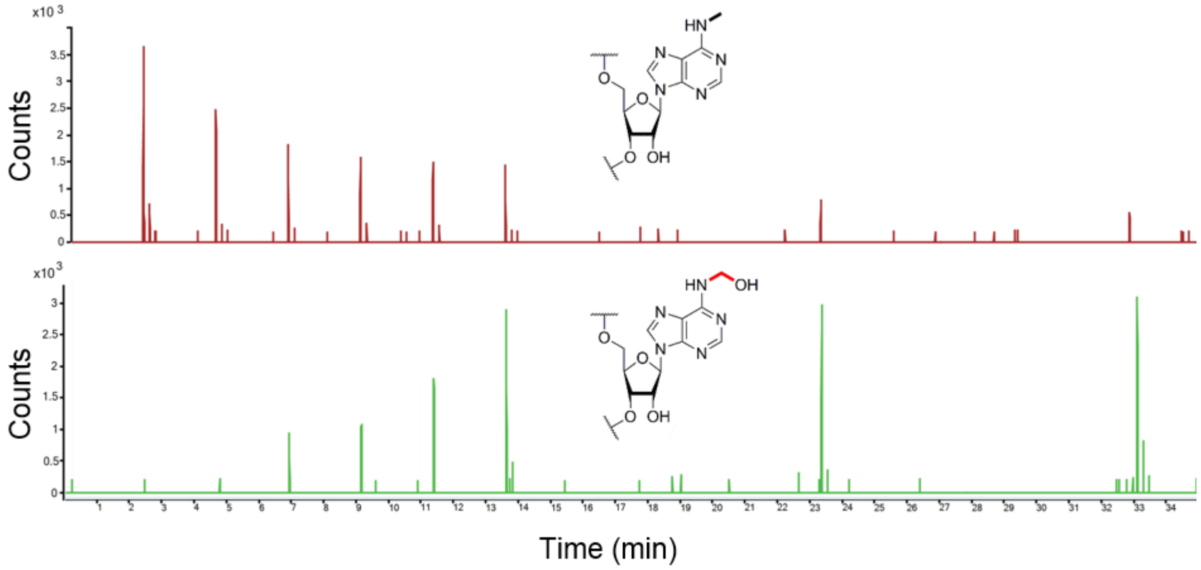

Figure S21. Graphs showing time-dependent formation of (6-hydroxymethyl)adenine (6hmA, green) after incubation of an RNA oligonucleotide containing (6-methyl)adenine (m6A, red) with FTO, as determined by MS analysis. No evidence for the un-methylated oligonucleotide was accrued during the time course. For experimental details, see below.

EXPERIMENTAL

Materials

Adenosine, adenosine monophosphate, adenosine diphosphate, adenosine triphosphate, deoxyadenosine monophosphate, cytidine, deoxycytidine, deoxycytidine monophosphate, deoxyguanosine monophosphate, thymidine monophosphate, (5-hydroxymethyl)deoxycytidine, (1-methyl)adenosine, (6-methyl)adenosine, (5-methyl)deoxycytidine, and uridine monophosphate were from Sigma Aldrich. Recombinant FTO protein was produced in *Escherichia coli* as reported.[1] Procedures for the synthesis of m6A phosphoramidite, (6-^{13}C-methyl)adenosine and (3-^{13}C-methyl) thymidine, used in the experiments with FTO, are given in the Supplementary Information. Ammonium formate buffer was prepared as reported.[2] All other reagents were from Sigma Aldrich.

NMR Experiments

NMR experiments were carried out using either a Bruker AVII 500 spectrometer equipped with a TXI probe, a Bruker AVIII 600 spectrometer equipped with a Prodigy N2 broadband cryoprobe, or a Bruker AVIII 700 spectrometer equipped with an inverse TCI ^{1}H/^{13}C/^{15}N cryoprobe. All spectrometers were operated using TOPSPIN 3 software. ^{1}H chemical shifts are reported in ppm relative to the solvent resonance (δ_H 4.7 ppm), while signal intensities were calibrated relative to 3-(trimethylsilyl)-2,2,3,3-tetradeuteropropionic acid (TSP, δ_H 0 ppm), which was added to each sample. The deuterium signal of D_2O was used as an internal lock signal.

Samples containing nucleotides and HCHO were prepared by mixing stocks of nucleotides in D_2O (pD adjusted) with HCHO in D_2O. TSP was added before transferring to either 3 mm or 5 mm NMR tubes for analysis by ^{1}H NMR. The total lapse time between mixing and data acquisition was 5-7 minutes. Concentrations of products were quantified relative to the concentration of added TSP (5.8 mM). 2-Dimensional heteronuclear single quantum correlation (2D-^{1}H-^{13}C-HSQC) experiments and 2-dimensional heteronuclear multiple-bond correlation (2D-HMBC) experiments used standard Bruker pulse programs; for the HMBC analyses, the coupling evolution delay was 62.5 milliseconds. 25-Fold dilution experiments shown in **Main Text Figure 2** were carried out by diluting the reaction mixture containing the nucleotides (2.4 mM) and HCHO (53 equivalents) with D_2O after one week incubation. Dilution experiments shown in **Figure S4** (2-25-fold dilutions) were carried out by pre-incubating stock solutions of nucleotides (10 mM) and HCHO (8 equivalents) for one week, before splitting the stock solutions and diluting with the appropriate quantities of D_2O.

EXSY analyses were carried out using a 1-dimensional gradient-selected NOESY pulse sequence employing selective refocusing with a Gaussian pulse.[3-5] Experiments were run accumulating 16 transients with mixing times (τm) of 10–1200 ms, with the ^{1}H-resonance of hydrated HCHO (δ_H 4.89 ppm at 37 °C) being selectively irradiated. Adduct formation rates were calculated as follows: the normalised intensities of the EXSY correlations (i.e. the intensities of the ^{1}H-resonances corresponding to the adduct *N*-hydroxymethyl protons normalised to the intensity of the irradiated HCHO resonance) were plotted as a function of τm, and the initial intensity build-up rates were determined (see **Figure S11**). Assuming a bimolecular mechanism for adduct formation, the build-up rates represent $k_1[\text{nucleotide}_{eq}]$, where k_1 is the rate constant for adduct formation, and $[\text{nucleotide}_{eq}]$ is the concentration of unreacted nucleotide at equilibrium (see below). The initial adduct formation rate for 3hmUMP and 3hmTMP were calculated by dividing their accumulation rate by $[\text{nucleotide}_{eq}]$ and then multiplying by the initial nucleotide concentration ([nucleotide], 2.4 mM) and initial HCHO concentration ([HCHO]). The unimolecular hydrolysis rate constants (k_{-1}) were calculated by noting that, at equilibrium, formation and hydrolysis rates are equal, i.e. $k_1[\text{nucleotide}_{eq}][\text{HCHO}_{eq}] = k_{-1}[\text{adduct}_{eq}]$, where $[\text{HCHO}_{eq}]$ is the concentration of HCHO at

equilibrium and [adduct_{eq}] is the concentration of adduct at equilibrium. [HCHO_{eq}] = [HCHO] – [adduct_{eq}].

$$\text{Nucleotide + HCHO} \underset{k_{-1}}{\overset{k_1}{\rightleftharpoons}} \text{Adduct}$$

Reaction scheme showing adduct formation via a bimolecular mechanism.

FTO catalysis was monitored using both ^{1}H NMR and the gradient-selected 1 dimensional heteronuclear single quantum correlation (^{1}H-^{13}C-HSQC) method. Samples were prepared containing FTO (prepared as reported[1], with a final concentration of 20 µM), the nucleoside selectively ^{13}C-labelled of the methyl group (400 µM), 2-oxoglutarate (2OG, 5 mM), sodium ascorbate (1 mM), ferrous iron (20 µM) in ammonium formate buffer in D_2O pH* 7.5. The samples were then transferred to 3 mM MATCH NMR tubes (Hilgenberg) and monitored by NMR. ^{1}H analyses employed NOESY water presaturation, while the 1 dimensional heteronuclear single quantum correlation (1D-^{1}H-^{13}C-HSQC) method was derived from the standard 2D ^{1}H-^{13}C-HSQC pulse sequence to remove both the variable t_1 period and ^{13}C decoupling during data acquisition. The $1/2J_{CH}$ delays were optimised for 145 Hz.

MS-Based FTO Activity Assay

A reaction mixture containing RNA oligonucleotide (AUUGUGG-m6A-CUGCAGC, 1 µM), 2OG (10 µM), ascorbate (100 µM), ferrous iron (10 µM) and FTO (100 nM) in 50 mM Tris buffer in H_2O at pH 7.5 was prepared in a 2 mL 96-well plate (Greiner) and reaction progression was monitored by MS using an Agilent RapidFire RF360 high throughput system paired with an Agilent quad time of flight (Q-TOF) mass spectrometer. Aliquots from the mixture were periodically subjected to MS analysis (at 1 minute intervals over the first 14 minutes after mixing, then at 23 minutes and 33 minute after mixing). The sample was passed through an Agilent C8 RapidFire cartridge, which isolated the oligonucleotides; the oligonucleotides were then eluted from the cartridge with 600mM octyl ammonium acetate (OAA) (20 %) and acetonitrile (80 %), and injected into the spectrometer. The RapidFire RF360 system was operated using RapidFire RF360 integrated software, and the mass spectrometer was operated using Agilent MassHunter Workstation Data Acquisition software. Signal intensities were quantified as the total ion count, and analysed using RapidFire integrator software (Agilent). Detailed RapidFire-MS procedures will be published elsewhere.

CHEMICAL SYNTHESIS

General Methods

All chemicals, including dried solvents, were from Sigma-Aldrich and used without further purification. Solvents used for work-up and chromatography were from Aldrich at HPLC grade. Silica gel 60 F254 analytical thin layer chromatography (TLC) plates were from Merck. Pre-packed SNAP columns were used for chromatography on a Biotage SP1 Purification system. Proton and Carbon NMR spectra were acquired using AVIIIHD 500 or Bruker AVIIIHD 400 or AVIIIHD 600 with N_2 cryoprobe. Shifts are reported in δ ppm. Abbreviations s, d, t, q, and m denoting singlet, doublet, triplet, quartet and multiplet respectively used in ^{1}H NMR. Coupling constants, *J*, are registered in Hz to a resolution of 0.5 Hz. High Resolution (HR) mass spectrometry data (m/z) were obtained from a Bruker MicroTOF instrument using an ESI source and Time of Flight (TOF) analyzer. Values are reported as ratio of mass to charge in Daltons. Melting points were obtained using a Leica VMTG heated-stage microscope or Stuart SMP-40 automatic melting point apparatus. All reactions were carried out in an oven-dried round bottom flask. A magnetic stirrer was used to ensure homogenous mixing during the reaction. Synthesis of oligonucleotides were carried out as reported.[6]

Synthesis of Synthesis of (3-(^{13}C)methyl)thymidine

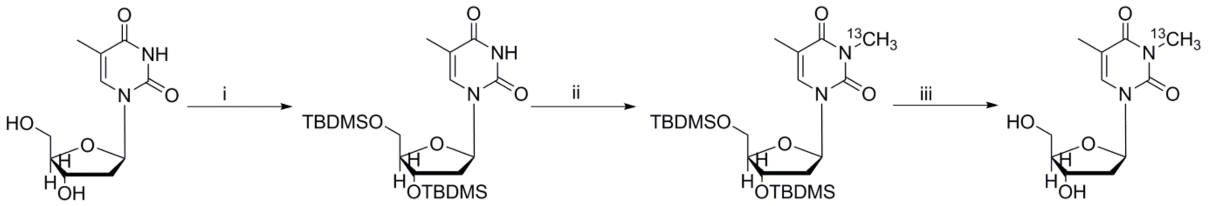

Scheme S1. Synthetic route to (3-(^{13}C)methyl)thymidine. i) Imidazole, TBDMSCl, DMF, 4 hr; ii) NaH, DMF, $^{13}CH_3I$; iii) $3HF.NEt_3$, CH_2Cl_2.

3', 5'-*O*-Bis(*t*-butyldimethylsilyl)thymidine

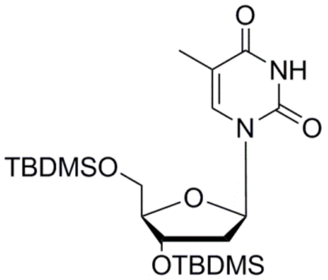

This compound was synthesised using a reported procedure.[7] To a stirred solution of 2'-deoxythymidine (2 g, 8.3 mmol) and imidazole (2.25 g, 33.026 mmol) in dry DMF in a 50 mL round bottom flask was added TBDMSCl (2.74 g, 18.16 mmol) portionwise. The reaction was stirred for four hours. The resulting mixture was concentrated under reduced pressure, then diluted with EtOAc. The EtOAc layer was washed with water (3 x 10 mL), and finally with brine. The organic layer was dried over $MgSO_4$ and concentrated in *vacuo*. Mixture was purified (19:1 to 4:1 cyclohexane / EtOAc) by column chromatography which resulted in an off-white solid (3.3 g, 86%). m.p.: 150.8 °C.TLC R_f 0.15 (4:1 cyclohexane / EtOAc). ^{1}H NMR (600 MHz, $CDCl_3$) δ 0.10 (s, 3H), 0.11 (s, 3H), 0.14 (s, 3H), 0.14 (s, 3H), 0.92 (s, 9H), 0.96 (s, 9H), 1.94 (d, *J* = 1.0 Hz, 3H), 2.03 (ddd, *J* = 13.0, 8.0, 6.0 Hz, 1H), 2.27 (ddd, *J* = 13.0, 6.0, 2.5 Hz, 1H), 3.79 (dd, *J* = 11.5, 2.5 Hz, 1H), 3.89 (dd, *J* = 11.5, 2.5 Hz, 1H), 3.96 (q, *J* = 2.5 Hz, 1H), 4.43

(dt, J = 6.0, 2.5 Hz, 1H), 6.35 (dd, J = 8.0, 6.0 Hz, 1H), 7.49 (d, J = 1.0 Hz, 1H), 8.15 (b.s, 1H). ^{13}C NMR (151 MHz, $CDCl_3$) δ -5.4, -5.35, -4.8, -4.6, 12.5, 18.0, 18.4, 25.8, 25.9, 41.4, 63.0, 72.3, 84.9, 87.9, 110.8, 135.5, 150.1, 163.4. HRMS (ESI) m/z: Calculated for $C_{22}H_{43}O_5N_2Si_2$ $[M+H]^+$ 471.2705. Observed: 471.2697.

3', 5'-*O*-Bis(*t*-butyldimethylsilyl)-*N3*-(^{13}C)methylthymidine

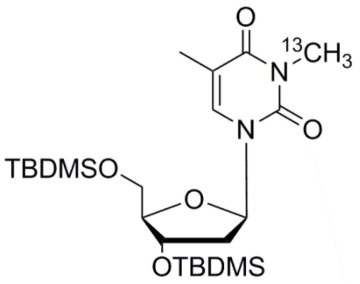

3', 5'-O-Bis(*t*-butyldimethylsilyl)-2'-deoxythymidine (0.8 g, 1.7 mmol) was dissolved in anhydrous DMF at 0°C under a nitrogen atmosphere. NaH (0.072 g, 1.78 mmol) was dissolved in DMF; this solution was carefully added and the resultant mixture was stirred for 1 hr to give a clear solution. ^{13}C-methyl iodide was added dropwise and the solution was stirred overnight. The solvent was removed under reduced pressure. The reduced mixture was diluted with EtOAc and washed subsequently with water and brine. The organic layer was dried over $MgSO_4$ and concentrated under reduced pressure. The mixture was purified (19:1 to 4:1 cyclohexane / EtOAc) by column chromatography which resulted in a white solid (0.2 g, 24 %). TLC R_f 0.2 (4:1 cyclohexane / EtOAc). ^{1}H NMR (600 MHz, $CDCl_3$) δ 0.10 (s, 3H), 0.11 (s, 3H), 0.13 (s, 3H), 0.14 (s, 3H), 0.92 (s, 9H), 0.95 (s, 9H), 1.96 (d, J = 1.0 Hz, 3H) 2.02 (ddd, J = 13.0, 8.0, 6.0 Hz, 1H), 2.29 (ddd, J = 13.0, 6.0, 2.5 Hz, 1H), 3.25,3.48 (s, 3H), 3.79 (dd, J = 11.5, 2.5 Hz, 1H), 3.89 (dd, J = 11.5, 2.5 Hz, 1H), 3.96 (q, J = 2.5 Hz, 1H), 4.38 – 4.45 (m, 1H), 6.35 (dd, J = 8.0, 6.0 Hz, 1H),), 7.49 (d, J = 1.0 Hz, 1H). ^{13}C NMR (151 MHz, $CDCl_3$) δ -5.4, -5.37, -4.8, -4.6, 13.3, 18.0, 18.4, 25.8, 25.9, 27.8, 41.5, 63.0, 72.2, 85.6, 87.9, 109.9, 133.3, 151.1, 163.7. HRMS (ESI) m/z: Calculated for $C_{10}{}^{13}CH_{17}O_5N_2Na$ [M+Na]+ 508.2715. Observed: 508.2708.

(3-(^{13}C)-Methyl)thymidine

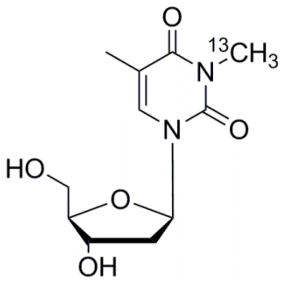

This compound was prepared according to a modified version of a reported procedure.[8] To a stirred solution of 3', 5'-*O*-Bis(*t*-butyldimethylsilyl)-*N3*-(^{13}C)methylthymidine (0.13 g, 0.3 mmol) in CH_2Cl_2 was added 3HF.NEt_3 (100 µL, 0.5 mmol) dropwise; the resultant reaction mixture was stirred for 48 hr. The solution was then concentrated under reduced pressure and purified (99:1 to 20:3 EtOAc / MeOH) by column chromatography to give a white solid (0.05g, 75.7%). TLC R_f 0.20. ^{1}H NMR (600 MHz, D_2O) δ 1.87 (b.s, 3H), 2.26-2.37 (m, 2H), 3.12 ,3.36 (s, 3H), 3.71 (dd, J = 12.5, 3.5 Hz, 1H), 3.79 (dd, J = 12.5, 3.5 Hz, 1H), 3.97 (q, J = 3.5 Hz, 1H) 4.2-4.46 (m, 1H), 6.27 (dd, J = 8.0, 6.0 Hz, 1H), 7.60 (s, 1H). ^{13}C NMR (151 MHz, D_2O) δ 12.4,

38.2, 27.9, 61.0, 86.6, 70.8, 85.8, 135.4, 110.4, 152.2, 163.7. HRMS (ESI) m/z: Calculated for $C_{10}{}^{13}CH_{17}O_5N_2$ [M+H]+ 258.1166. Observed: 258.1167.

Synthesis of (6-(^{13}C)methyl)adenosine

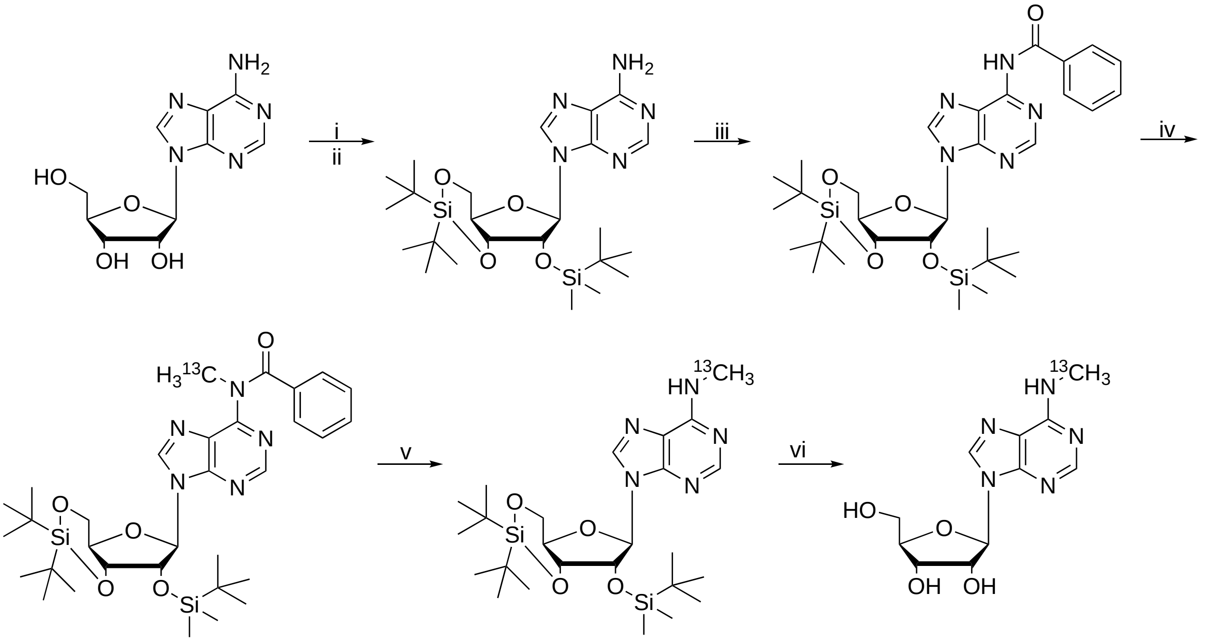

Scheme S2. Route to (6-(^{13}C)methyl)adenosine. i) (*t*-Bu)$_2$Si(OTf)$_2$, DMF, imidazole, 0 °C,1 hr; ii) TBDMSCl, DMF, imidazole, 60 °C,12 hr; iii) PhCOCl, pyridine, 0-20 °C,3 days; iv) NH_3/MeOH, 23 °C; v) $^{13}CH_3I$, NaOH, CH_2Cl_2, tetrabutylammonium Iodide, 24 hr; vi) LiBHEt$_3$, DMF,1 hr; vii) 3HF.NEt$_3$, CH_2Cl_2, 24 hr.

3',5'-*O*-Bis(*t*-butylsilyl)-2'-*O*-(*t*-butyldimethylsilyl)adenosine

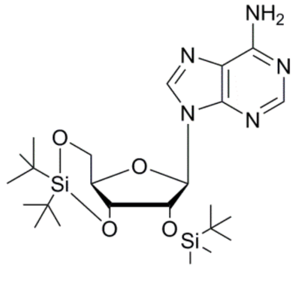

This compound was prepared according to a modified version of a reported procedure.[9] To a stirred suspension of adenosine (2.12 g, 8 mmol) in 40 mL anhydrous DMF at 0 °C, di-*t*-butylsilyl ditrifluoromethanesulfonate (3.87 mL, 8.8 mmol) was added drop wise under an N_2 atmosphere. After consumption of starting material (30 min, as assessed by TLC), the reaction was quenched immediately with imidazole (2.7 g, 40 mmol) at 0 °C. After 5 minutes, the reaction was warmed to room temperature. Then, *t*-butyldimethylsilyl chloride (1.45 g, 9.6 mmol) was added portion wise and the reaction was refluxed at 60 °C for 12 hr. The suspension was cooled down to room temperature, water was added and the precipitate was collected by suction filtration. The filtrate was discarded, and the white precipitate was washed with cold MeOH. The MeOH layer was evaporated under reduced pressure and the product was crystallised from CH_2Cl_2 to give a white solid (3.8 g, 91%). m.p 191-193.4°C. TLC R_f 0.45 (3:2 cyclohexane / EtOAc). ^{1}H NMR (400 MHz, $CDCl_3$) δ 0.00 (s, 3H), 0.02 (s, 3H), 0.78 (s,

9H), 0.90 (s, 9H), 0.94 (s, 9H), 3.89 (dd, J = 10.5, 5.0 Hz, 1H), 4.07 (dd, J = 10.5, 9.0 Hz, 1H), 4.34 (dd, J = 9.0, 5.0 Hz, 1H), 4.40 (dd, J = 9.0, 4.5 Hz, 1H), 4.48 (d, J = 4.5 Hz, 1H), 5.36 (b.s, 2H), 5.76 (b.s, 1H), 7.68 (s, 1H), 8.18 (s, 1H). ^{13}C NMR (100 MHz, $CDCl_3$) δ -6.3, -6.0, 16.9, 20.4, 22.8, 24.6, 27.0, 27.5, 62.4, 70.5, 74.5, 75.7, 83.0, 126.9, 143.6, 152.3, 154.4, 157.5. HRMS (ESI) m/z: Calculated for $C_{24}H_{44}O_4N_5Si_2$ [M+H]+ 522.2926. Observed: 522.2923.

3',5'-*O*-Bis(*t*-butylsilyl)-2'-*O*-(*t*-butyldimethylsilyl)-*N6*-benzoyladenosine

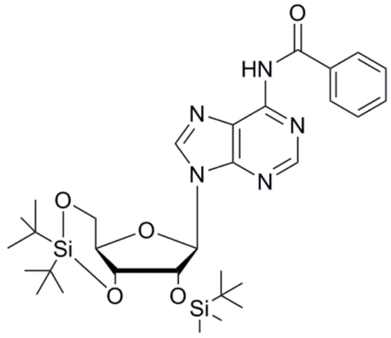

This compound was prepared according to a modified version of a reported procedure.[9] To a stirred solution of 3',5'-*O*-bis(*t*-butylsilyl)-2'-*O*-(*t*-butyldimethylsilyl)adenosine (2.08 g, 4 mmol) in 10 mL anhydrous pyridine at -5 °C (salt-ice bath), benzoyl chloride (0.93 mL, 8 mmol,) was added drop wise. The reaction mixture was allowed to warm to room temperature for 1 hour, and stirred further for 18 hours under N_2. The reaction was quenched first with 6 mL MeOH at 0 °C and then with excess methanolic ammonia (1 mL, 7 N NH_3) after 1 hr. The crude product mixture was concentrated under pressure, and was then diluted with EtOAc and saturated $NaHCO_3$ solution. The organic layer was washed with water (3 x 10 mL) and dried with anhydrous $MgSO_4$ and reduced under vacuum. The mixture was purified (6:1 to 3:1 cyclohexane / EtOAc) by column chromatography resulting in an off-white solid (2.2 g, 88%). mp 127-128 °C TLC Rf 0.35 (2:3 cyclohexane / EtOAc). ^{1}H NMR (400 MHz, $CDCl_3$) δ 0.00 (s, 3H), 0.02 (s, 3H), 0.78 (s, 9H), 0.89 (s, 9H), 0.92 (s, 9H), 3.88 (dd, J = 10.5, 9.0 Hz, 1H), 4.09 (dd, J = 10.5, 5.0 Hz, 1H), 4.33 (ddd, J = 5.0, 9.0, 5.0 Hz, 1H), 4.47 (d, J = 5.0 Hz, 1H), 5.84 (s, 1H), 7.40 – 7.32 (m, 3H), 7.42 – 7.48 (m, 1H), 7.83 – 7.89 (m, 3H), 8.60 (s, 1H), 8.85 (s, 1H). ^{13}C NMR (100 MHz, $CDCl_3$) δ -5.0, -4.2, 18.3, 20.4, 21.1, 25.9, 27.0, 27.5, 67.8, 74.8, 75.5, 75.9, 92.5, 123.8, 127.9, 128.9, 132.9, 133.5, 141.1, 149.6, 151.2, 152.7, 164.6. HRMS (ESI) m/z: Calculated for $C_{31}H_{48}O_5N_5Si_2$ [M+H]+ 626.3188. Observed: 626.3184.

3',5'-*O*-Bis(*t*-butylsilyl)-2'-*O*-(*t*-butyldimethylsilyl)-*N6*,*N6*-benzoyl-(^{13}C)-methyladenosine

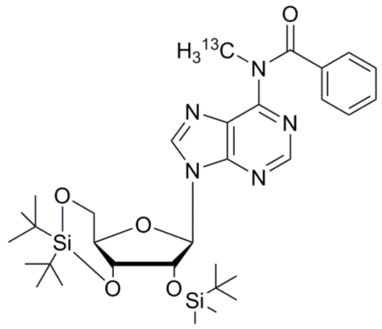

The compound was prepared according to a modified reported procedure.[10] To a solution of 3',5'-*O*-bis(*t*-butylsilyl)-2'-*O*-(*t*-butyldimethylsilyl)-*N6*-benzoyladenosine (260 mg, 0.42 mmol) in 5 mL CH_2Cl_2, tetrabutylammonium bromide (0.23 g, 1.6 mmol) and 1 N aq. NaOH (excess) were added. After 20 min, ^{13}C-methyl iodide (104 µL, 1.7 mmol) was added to the reaction

mixture and further stirred for 24 hr. Excess CH_2Cl_2 was added and the mixture was washed with water (3 x 10 mL). The organic phase was then dried ($MgSO_4$) and concentrated under reduced pressure. The residue was purified by column chromatography (4:1 to 3:2 cyclohexane / EtOAc) which resulted in an oil (140 mg, 52 %). TLC R_f 0.5 (7:3 cyclohexane / EtOAc). ^{1}H NMR (400 MHz, $CDCl_3$) δ -0.01 (s, 3H), -0.00 (s, 3H), 0.79 (s, 9H), 0.89 (s, 9H), 0.90 (s, 9H), 3.43, 3.77(s,3H) , 3.74 – 3.85 (m, 1H), 3.94 – 4.09 (m, 1H), 4.14 (dd, *J* = 9.0, 5.0 Hz, 1H), 4.27 (d, *J* = 5.0 Hz, 1H), 4.32 (dd, *J* = 9.0, 5.0 Hz, 1H), 5.70 (s, 1H), 7.23 – 7.31 (m, 2H), 7.31 – 7.43 (m, 1H), 7.55 (s, 1H), 7.82 (d, *J* = 2.5 Hz, 1H), 8.04 (s, 1H), 8.06 (s, 1H). ^{13}C NMR (100 MHz, $CDCl_3$) δ -5.0, -4.3, 18.3, 20.4, 22.8, 25.9, 27.0, 27.5, 35.9, 36.9, 67.8, 74.7, 75.8, 75.9, 91.9, 122.7, 128.1, 129.8, 132.0, 135.7, 137.7, 144.7, 146.0, 146.9, 147.7. HRMS (ESI) m/z: Calculated for $C_{31}{}^{13}CH_{50}O_5N_5Si_2$ [M+H]+ 641.3378. Observed: 641.3376.

3',5'-*O*-Bis(*t*-butylsilyl)-2'-*O*-(*t*-butyldimethylsilyl)-*N6*-(^{13}C)methyladenosine

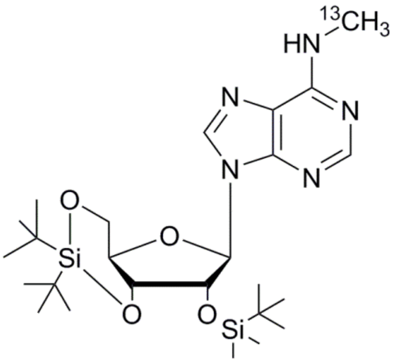

This compound was prepared according to a modified version of a reported procedure.[11] To a stirred solution of 3',5'-*O*-bis(*t*-butylsilyl)-2'-*O*-(*t*-butyldimethylsilyl)-*N6*,*N6*-benzoyl-(^{13}C)-methyladenosine (140 mg, 0.22 mmol) in THF (2 ml) at 0 °C, lithium triethylborohydride (1 M in THF, 1.1 mL, 1.09 mmol) was added and stirred for 1 hr at room temperature. The reaction was quenched by addition of saturated aqueous NH_4Cl, followed by 1 N NaOH. The crude product mixture was concentrated under vacuum, then diluted with EtOAc, then washed with water (3 x 10 mL). The organic layer was dried ($MgSO_4$) and concentrated under reduced pressure. The residue was purified by column chromatography (9:1 to 3:2 cyclohexane / EtOAc) which resulted in an oil (88 mg, 75%). TLC Rf 0.2 (7:3 cyclohexane / EtOAc). ^{1}H NMR (400 MHz, $CDCl_3$) δ 0.00 (s, 3H), 0.02 (s, 3H), 0.78 (s, 9H), 0.90 (s, 9H), 0.94 (s, 9H), 2.88, 3.23 (s, 3H), 3.89 (dd, *J* = 10.5, 9.0 Hz, 1H), 4.01 – 4.11 (m, 1H), 4.34 (dd, *J* = 9.0, 5.0 Hz, 1H), 4.41 (dd, *J* = 9.0, 5.0 Hz, 1H), 4.47 (d, *J* = 5.0 Hz, 1H), 5.76 (s, 2H), 7.62 (s, 1H), 8.22 (s, 1H). ^{13}C NMR (100 MHz, $CDCl_3$) δ -5.0, -4.3, 18.3, 20.4, 22.8, 25.9, 27.1, 27.5, 67.9, 74.7, 75.5, 75.8, 92.4, 120.6, 138.0, 153.4, 155.5. HRMS (ESI) m/z: Calculated for $C_{24}{}^{13}CH_{46}O_4N_5Si_2$ $[M+H]^+$ 537.3116. Observed: 537.3117.

(6-(^{13}C)-Methyl)adenosine

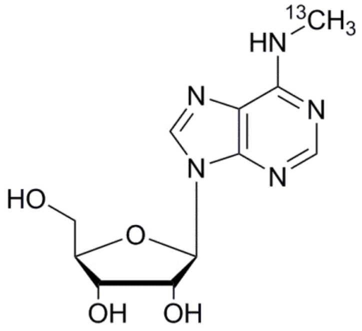

This compound was prepared according to a modified version of a reported procedure.[8] To a stirred solution of 3',5'-*O*-bis(*t*-butylsilyl)-2'-*O*-(*t*-butyldimethylsilyl)-*N6*-(^{13}C)methyladenosine (100 mg, 0.19 mmol) in CH_2Cl_2 was added 3HF.NEt_3 (303 µL, 1.86 mmol) dropwise. The solution was then stired for 48 hr. The mixture was reduced under pressure and was purified by column chromatography (99:1 to 9:1 MeOH / EtOAc) to give a white solid (40 mg, 76%). m.p. 214.5°C; Rf 0.4 (9:1 EtOAc / MeOH). ^{1}H NMR (400 MHz, MeOH-d_4) δ 2.95 (s, 2H), 3.22 – 3.49 (m, 5H), 3.77 (dd, *J* = 12.0 Hz, 1H), 3.91 (dd, *J* = 12.0 Hz, 1H), 4.13 – 4.23 (m, 1H), 4.25 – 4.44 (m, 1H), 4.71 – 4.80 (m, 1H), 5.97 (d, *J* = 6.0 Hz, 1H), 8.24 (s, 2H). ^{13}C NMR (100 MHz, CH_3OD-d_4) δ 26.3, 62.1, 71.3, 74.1, 86.8, 89.9, 120.2, 140.0, 147.5, 152.1, 155.5. HRMS (ESI) m/z: Calculated for $C_{10}{}^{13}CH_{16}O_4N_5$ $[M+H]^+$ 283.1230. Observed: 283.1232.

Synthesis of m6A phosphoramidite

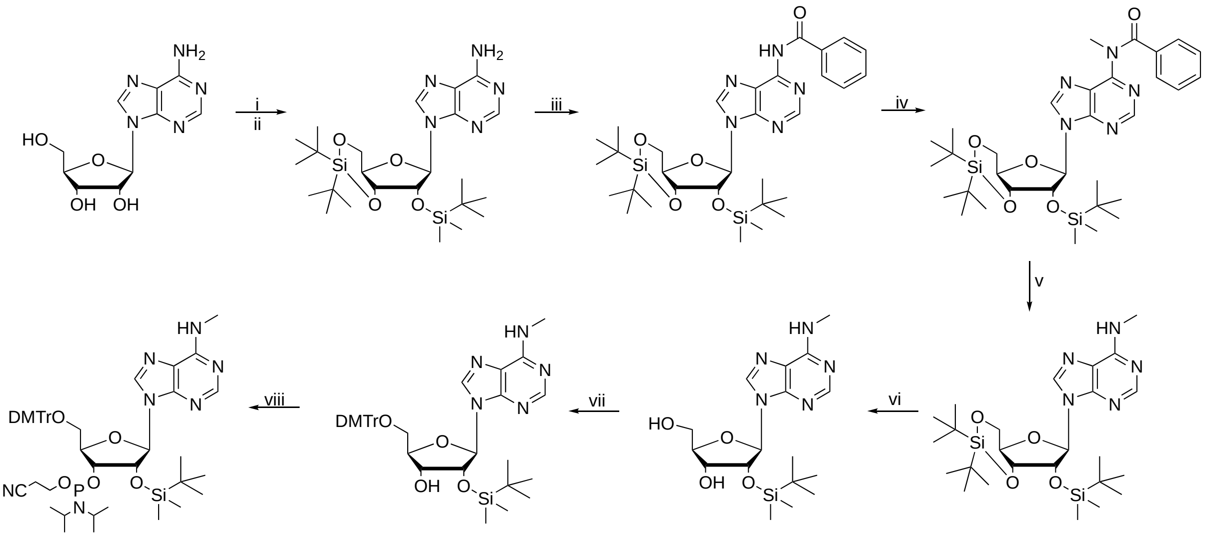

Scheme S3. Route to m6A phosphoramidite. i) (*t*-Bu)$_2$Si(OTf)$_2$, DMF, imidazole, 0 °C,1 hr; ii) TBDMSCl, DMF, imidazole, 60°C,12 hr; iii) PhCOCl, pyridine, 0-20 °C, 3 days, then NH_3/MeOH, 23 °C; iv) CH_3I, NaOH, CH_2Cl_2, tetrabutylammonium Iodide, 24 hr; v) $LiBHEt_3$, DMF,1 hr; vi) HF.pyridine (3 eq), pyridine, 0°C, 5 hr; vii) DMTrCl (1.2 eq), pyridine, 0°C, 12hr; viii) 2-cyanoethyl *N,N*-diisopropylchlorophosphoramidite (2.5 eq), diisopropylethylamine (DIPEA, 10 eq), THF, 0°C-rt, 6 hr.

3',5'-*O*-Bis(*t*-butylsilyl)-2'-*O*-(*t*-butyldimethylsilyl)-*N6*,*N6*-benzoylmethyladenosine

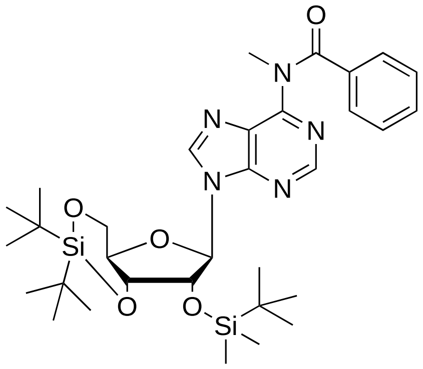

The compound was prepared according to a version of a reported procedure.[10] To a solution of 3',5'-*O*-Bis(*tert*-butylsilyl)-2'-*O*-(*tert*-butyldimethylsilyl)-*N*[6]-benzoyladenosine (1 g, 1.6 mmol) in 5 mL CH_2Cl_2, tetra-butylammonium bromide (0.88 g, 2.4 mmol) and 1 N aq. NaOH (excess) were added. After 20 min, methyl iodide (0.4 mL, 6.4 mmol) was added to the reaction mixture and further stirred for 24 hr. Excess CH_2Cl_2 was added to the residue and washed with water (3x10ml). The organic phase was dried ($MgSO_4$) and concentrated under vacuum. The residue was purified by column chromatography (4:1 to 3:2 cyclohexane / EtOAc) to give an oil (663 mg, 65%). TLC R_f 0.5 (7:3 C_6H_{12} /EtOAc); ^{1}H NMR (400 MHz, $CDCl_3$) δ 0.00 (s, 3 H) 0.02 (s, 3 H) 0.78 (s, 9 H) 0.90 (s, 9 H) 0.94 (s, 9 H) 3.05 (s, 3 H) 3.74 – 3.85 (m, 1H), 3.94 – 4.09 (m, 1H), 4.14 (dd, *J* = 9.0, 5.0 Hz, 1H), 4.27 (d, *J* = 5.0 Hz, 1H), 4.32 (dd, *J* = 9.0, 5.0 Hz, 1H), 5.70 (b.s, 1H), 7.23 – 7.31 (m, 2H), 7.31 – 7.43 (m, 1H), 7.55 (s, 1H), 7.82 (d, *J* = 2.5 Hz, 1H), 8.04 (s, 1H), 8.06 (s, 1H); ^{13}C NMR (100 MHz, $CDCl_3$) δ -5.0, -4.3, 18.3, 20.4, 21.1, 25.9, 27.0, 27.5, 35.8, 67.7, 74.7, 75.5, 75.8, 92.4, 126.7, 127.9, 128.7, 130.7, 136.0, 141.3, 151.7, 152.0, 155.0, 172.2; HRMS (ESI) m/z: Calculated for $C_{32}H_{50}O_5N_5{}^{28}Si_2$ $[M+H]^+$ 640.3345, Observed: 640.3331.

3',5'-*O*-Bis(*tert*-butyl)silyl-2'-*O*-(*tert*-butyldimethyl)silyl-*N*[6]-methyladenosine

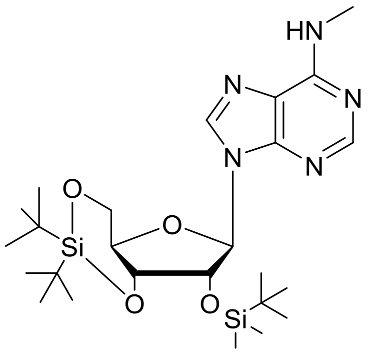

This compound was prepared according to a modified version of a reported procedure.[11] To a stirred solution of the 3',5'-*O*-Bis(*tert*-butylsilyl)-2'-*O*-(*tert*-butyldimethylsilyl)-*N*[6],*N*[6]-benzoylmethyladenosine (663 mg, 1.04 mmol) in THF (5.2 mL) at 0°C lithium triethylborohydride (1 M in THF, 4.15 mL, 4.15 mmol) was added and stirred for 1 hr at room temperature. The reaction was quenched by addition of saturated aqueous NH_4Cl followed by 1 N NaOH. The crude product mixture was concentrated under reduced pressure and then diluted with EtOAc and was washed with water (3 x 10mL). The organic layer was dried ($MgSO_4$) and concentrated under vacuum. The residue was purified by column

chromatography (9:1 to 3:2 cyclohexane / EtOAc) to give an oil (240 mg, 43 %). TLC R_f 0.20 (7:3 cyclohexane / EtOAc); ^{1}H NMR (400 MHz, $CDCl_3$) δ 0.00 (s, 3 H) 0.02 (s, 3 H) 0.78 (s, 9 H) 0.90 (s, 9 H) 0.94 (s, 9 H) 3.05 (d, *J* = 1.0, 3 H) 3.86-3.90 (m, 1 H) 4.02 - 4.10 (m, 1 H) 4.34 (dd, *J* = 9.0, 5.0, 1 H) 4.38 - 4.44 (m, 1 H) 4.47 (d, *J* = 4.5, 1 H) 5.76 (b.s, 2 H) 7.62 (s, 1 H) 8.22 (s, 1 H); ^{13}C NMR (101 MHz, $CDCl_3$) δ -5.0, -4.3, 18.3, 20.4, 22.8, 25.9, 27.1, 27.5, 27.6, 67.9, 74.6, 75.5, 75.8, 92.4, 127.6, 128.5, 138.0, 153.4, 155.5; HRMS (ESI) m/z: Calculated for $C_{25}H_{46}O_4N_5{}^{28}Si_2$ $[M+H]^+$ 536.3082, Observed: 536.3078.

2'-*O*-(*tert*-Butyldimethyl)silyl-*N*⁶-methyladenosine

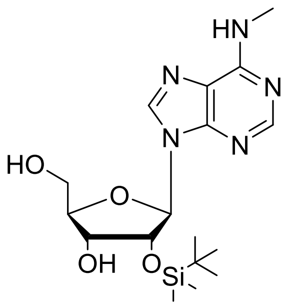

This compound was prepared according to a reported procedure.[12] To a stirred solution of 3',5'-*O*-Bis(*tert*-butylsilyl)-2'-*O*-(*tert*-butyldimethylsilyl)-*N*^{6},*N*^{6}-benzoylmethyladenosine (240 mg, 0.47 mmol) in 4mL of CH_2Cl_2 at -15°C was added cooled solution of (HF)x.py (0.06 mL, 2.33 mmol) diluted with 365 μL pyridine. The reaction temperature was maintained at -15 °C and allowed to stir for 12 hr. The reaction was diluted with CH_2Cl_2 and was washed first with sat. aq. $NaHCO_3$ solution then with water (3 x 10 mL). The organic layer was dried (anhydrous $MgSO_4$) and concentrated under reduced pressure. The residue was purified by column chromatography (9:1 to 3:2 cyclohexane / EtOAc) to give an oil (93 mg, 53%). TLC R_f 0.15 (2:3 hexane / EtOAc); ^{1}H NMR (400 MHz, $CDCl_3$) δ 0.00 (s, 3H), 0.02 (s, 3H), 0.78 (s, 9H), 0.90 (s, 9H), 0.94 (s, 9H), 3.42 (d, *J* = 1.0, 3 H), 3.89 (dd, *J* = 10.5, 9.0 Hz, 1H), 4.01 – 4.11 (m, 1H), 4.34 (dd, *J* = 9.0, 5.0 Hz, 1H), 4.41 (dd, *J* = 9.0, 5.0 Hz, 1H), 4.47 (d, *J* = 5.0 Hz, 1H), 5.76 (s, 1H), 7.62 (s, 1H), 8.22 (s, 1H); ^{13}C NMR (100 MHz, $CDCl_3$) δ -5.4, -5.3, 17.92, 25.6, 27.5, 63.5, 73.1, 74.4, 87.8, 91.3, 119.7, 127.6, 140.1, 152.9, 156.0; HRMS (ESI) m/z: Calculated for $C_{17}H_{30}O_4N_5{}^{28}Si$ $[M+H]^+$ 396.2062, Observed: 396.2068.

5'-*O*-(4,4'-Dimethoxytrityl)-2'-*O*-dimethyl(*tert*-butyl)silyl-*N*6-methyladenosine

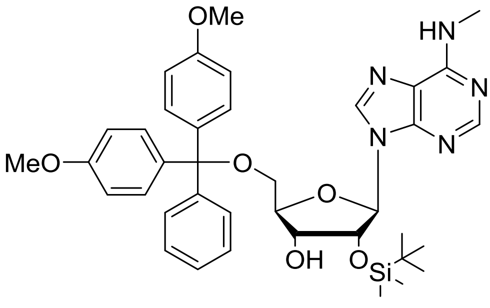

This compound was prepared according to a reported procedure.[12] To a stirred solution of 2'-*O*-dimethyl(*tert*-butyl)silyl-*N*6-methyladenosine (2.6 g, 6.4 mmol) in 4 mL anhydrous pyridine at 0°C, DMTrCl (2.5 g, 7.6 mmol) was added portion wise at regular intervals for 12 hr. The reaction was quenched by addition of an excess of anhydrous MeOH (0.5 mL) at room temperature. After 1 hr, the solution was concentrated under vacuum. The crude solid was first fractioned between aqueous $NaHCO_3$ and EtOAc, and then the organic layer was washed

with water (3 x 10 mL). The organic layer dried $MgSO_4$ and concentrated under vacuum. The residue was purified by column chromatography (9:1 to 3:2 cyclohexane / EtOAc) to give a yellow oil (3 g, 68 %). TLC R_f 0.45 (2:3 cyclohexane / EtOAc); ^{1}H NMR (400 MHz, $CDCl_3$) δ -0.13 (s, 3 H) 0.00 (s, 3 H) 0.86 (s, 9 H) 2.77 (d, *J* = 4.0, 1 H) 3.17 (s, 3 H) 3.36 - 3.43 (m, 1 H) 3.54 (dd, *J* = 10.5, 3.5, 1 H) 3.80 (s, 6 H) 4.27 (d, *J* = 3.5, 1 H) 4.33 - 4.37 (m, 1 H) 5.02 (t, *J* = 5.5, 1 H) 5.85 (d, *J* = 4.5, 1 H) 6.04 (b.s, 2 H) 6.83 (d, *J* = 9.0, 4 H) 7.18 - 7.28 (m, 3 H) 7.36 (d, *J* = 8.0, 4 H) 7.47 (dd, *J* = 8.5, 1.3, 2 H) 7.98 (s, 1 H) 8.35 (s, 1 H); ^{13}C NMR (100 MHz, $CDCl_3$) δ -6.3, -6.0, 16.9, 24.6, 27.5, 54.2, 62.4, 70.5, 74.5, 83.0, 85.5, 112.1, 125.9, 126.9, 127.1, 129.1, 134.6, 137.4, 143.6, 152.2, 154.4, 157.5; HRMS (ESI) m/z: Calculated for $C_{38}H_{48}O_6N_5{}^{28}Si$ $[M+H]^+$ 698.3368; Observed: 698.3359.

5'-*O*-(4,4'-Dimethoxytrityl)-(3'-*O*-[(2cyanoethyl)(*N*,*N*-diisopropylamino)phosphino]-2'-*O*-dimethyl(*tert*-butyl)silyl-*N*6-methyladenosine

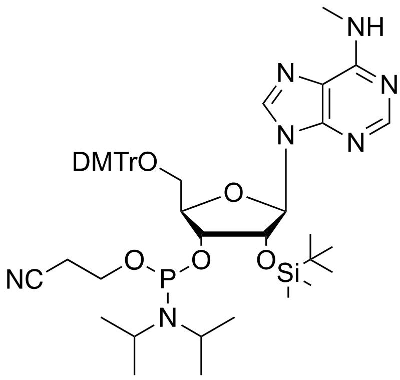

This compound was prepared according to a reported procedure.[12] To a stirred solution of 5'-*O*-(4,4'-Dimethoxytrityl)-2'-*O*-dimethyl(*tert*-butyl)silyl-*N*6-methyladenosine (500 mg, 0.71 mmol) in anhydrous CH_2Cl_2 in an over-dried flask under argon was added DIPEA (1.25 mL, 7.17 mmol) dropwise and the reaction mixture was allowed to stir at 0 °C for 10 min. (2-cyanoethyl)-*N,N*-diisopropylchlorophosphoramidite reagent (0.40 mL, 1.8 mmol) was added to the reaction mixture dropwise at 0 °C under under argon atmosphere. Reaction was stirred at 0 ° C for 30 minute and then gradually (about 30 minute) warmed up to room temperature. After another five hours under inert atmosphere, the reaction mixture was passed through saturated aq. KCl solution and was evaporated by rotary evaporation. The desired product was separated by silica gel column chromatography (1:1:0.01 hexane:EtOAc: pyridine) which resulted in colourless oil (400 mg, 62 %) yield. TLC R_f 0.40 (1:1:0.01 hexane:EtOAc: pyridine); ^{1}H NMR (700 MHz, CD_2Cl_2) δ -0.15 (s, 3H), -0.01 (s, 3H), 0.82 (s, 9H), 1.10 (s, 3H), 1.11 (s, 3H), 1.22 (s, 3H), 1.22 (s, 3H), 1.65 (s, 2H), 2.62 – 2.74 (m, 2H), 3.19 (s, 3H), 3.36 (dd, *J* = 10.5, 4.5 Hz, 1H), 3.54 (dd, *J* = 10.5, 4.0 Hz, 1H), 3.82 (s, 6H), 3.85-3.93 (m, 1H), 3.95-4.10 (m, 1H), 4.41 – 4.49 (m, 1H), 5.12 (dd, *J* = 6.1, 4.4 Hz, 1H), 5.33 – 5.40 (m, 2H), 5.79 (s, 1H), 5.99 (d, *J* = 6.0 Hz, 1H), 6.78 – 6.90 (m, 4H), 7.23 – 7.29 (m, 1H), 7.28 – 7.34 (m, 2H), 7.34 – 7.40 (m, 4H), 7.47 – 7.52 (m, 2H), 7.94 (s, 1H), 8.25 (s, 1H); ^{13}C NMR (176 MHz, CD_2Cl_2) δ -5.4, -5.0, 0.8, 17.8, 20.4, 24.4, 24.4, 25.4, 42.9, 43.0, 55.2, 55.2, 58.9, 63.5, 72.8, 72.9, 74.7, 74.7, 83.5, 86.5, 88.4, 113.1, 126.8, 127.8, 128.1, 129.0, 130.1, 130.1, 135.7, 139.0, 144.9, 153.0, 155.5, 158.7; ^{31}P NMR (202 MHz, CD_2Cl_2) δ 148.0, 150.8.

Synthesis of (6-methyl)guanosine

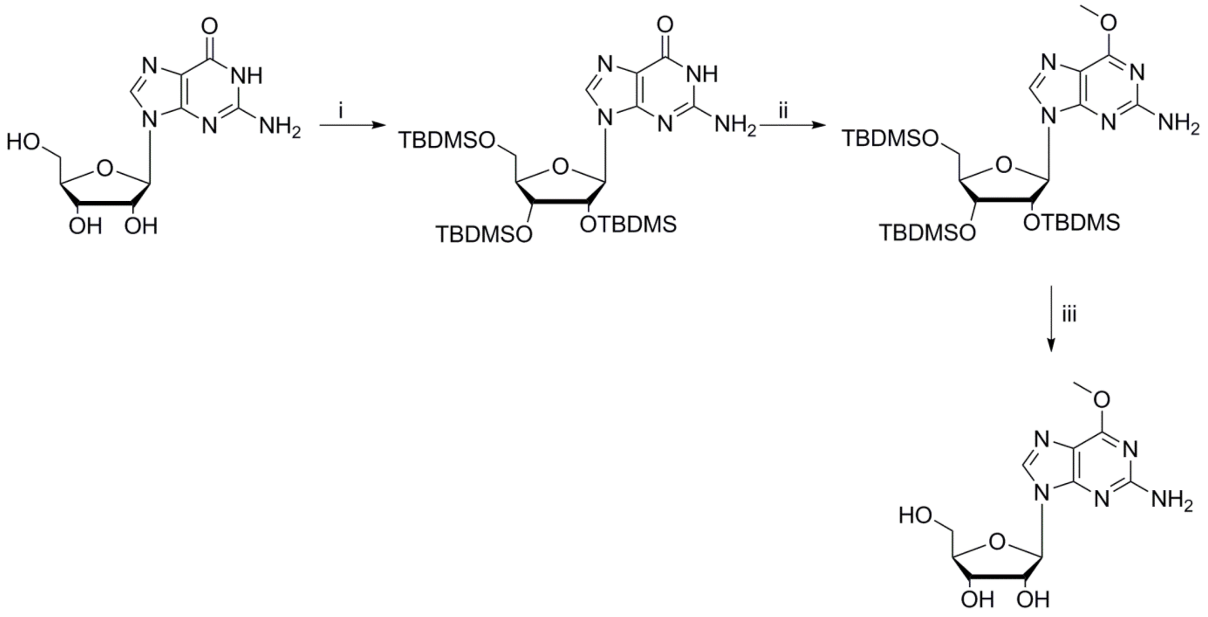

Scheme S3. Route to (6-methyl)guanosine. i) imidazole, TBDMSCl, DMF; ii) BOP, $CsCO_3$, MeOH; iii) $3HF.NEt_3$, CH_2Cl_2.

2', 3', 5'-*O*-tri(*t*-Butyldimethylsilyl)guanosine

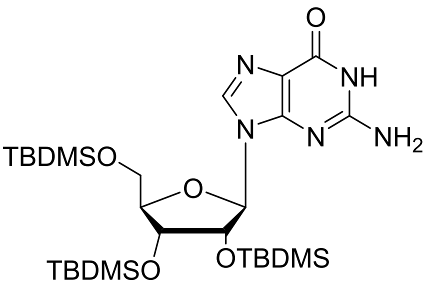

This compound was synthesized using a reported procedure.[13] To a stirred solution of guanosine (2 g, 8.3 mmol) and imidazole (3.75 g, 13.24 mmol) in dry DMF in a 50 mL round bottom flask was added TBDMSCl (6.58 g, 43.69 mmol) portion-wise. The reaction was stirred overnight. The resulting mixture was concentrated under reduced pressure and was diluted with EtOAc. The EtOAc layer was washed with water (3 x 10 mL) and finally with brine. The organic layer was dried over $MgSO_4$ and concentrated under reduced pressure. The mixture was purified (19:1 to 4:1 cyclohexane / EtOAc) by column chromatography resulting in an off-white solid (7.0 g, 84.5 %). TLC R_f 0.6 (9:1 CH_2Cl_2 / MeOH) m.p: 193.5°C. ^{1}H NMR (500 MHz, $CDCl_3$) δ -0.14 (s, 3H), -0.07 (s, 3H), 0.00 (s, 3H), 0.04 (s, 3H), 0.77 (s, 9H), 0.83 (s, 9H), 0.87 (s, 9H), 3.69 (dd, *J* = 11.5, 2.5 Hz, 1H), 3.84 – 3.93 (m, 1H), 4.00 (dt, *J* = 6.0, 2.5 Hz, 1H), 4.18 (b.s, 1H), 4.34 (b.s, 1H), 5.73 (b.s, 1H), 6.11 (b.s, 2H), 7.80 (s, 1H), 11.92 (s, 1H). ^{13}C NMR (126 MHz, $CDCl_3$) δ -5.3, -5.3, -4.8, -4.7, -4.2, 18.0, 18.1, 18.6, 25.8, 25.9, 26.2, 62.1, 71.2, 76.2,84.6, 88.2, 117.5, 136.1, 151.5, 153.5, 159.2. Calculated for $C_{28}H_{56}O_5N_5Si_3$ [M+H]+ 626.3584. Observed: 626.3576.

2', 3', 5'-*O*-tri(*t*-Butyldimethylsilyl)-*O*^{6}-methylguanosine

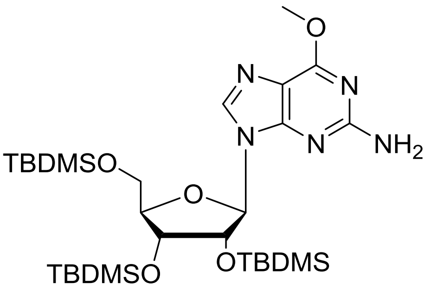

This compound was synthesised according to a reported procedure.[14] To a stirred solution of 2', 3', 5'-*O*-tri(*t*-butyldimethylsilyl)guanosine (0.8 g, 1.28 mmol) in dry THF was added $CsCO_3$ (0.85 g, 2.6 mmol) and BOP (1.2 g, 2.7 mmol). After one hour, the resulting mixture was concentrated under reduced pressure. To this concentrated mixture was added $CsCO_3$ (0.85 g, 2.6 mmol) and MeOH (25 mL). After 2 hr, the resulting mixture was reduced under pressure and was diluted with EtOAc. The organic layer was washed with water (2 x 10 mL), brine and dried ($MgSO_4$). The concentrated residue was purified by column chromatography (9:1 to 4:1 cyclohexane / EtOAc) which resulted in an oil (0.6 g, 73 %). TLC R_f 0.8 (9:1 CH_2Cl_2 / MeOH). ^{1}H NMR (600 MHz, $CDCl_3$) δ -0.13 (s, 3H), -0.00 (s, 3H), 0.13 (s, 6H), 0.15 (s, 3H), 0.15 (s, 3H), 0.84 (s, 9H), 0.95 (s, 9H), 0.97 (s, 9H), 3.80 (dd, *J* = 11.5, 2.5 Hz, 1H), 4.00 (dd, *J* = 11.5, 4.0 Hz, 1H), 4.09 (s, 3H), 4.10 – 4.14 (m, 1H), 4.32 (t, *J* = 4.0 Hz, 1H), 4.53 (t, *J* = 4.5 Hz, 1H), 4.95 (s, 2H), 5.93 (d, *J* = 5.0 Hz, 1H), 8.01 (s, 1H). ^{13}C NMR (151 MHz, $CDCl_3$) δ -5.4, -5.0, -4.7, -4.7, -4.3, 17.9, 18.1, 18.6, 25.7, 25.9, 26.1, 53.8, 62.5, 71.9, 76.1, 85.1, 87.9, 115.9, 138.0, 153.6, 159.2, 161.4. Calculated for $C_{29}H_{58}O_5N_5Si_3$ [M+H]+ 640.3740. Observed: 640.3728.

(6-Methyl)guanosine

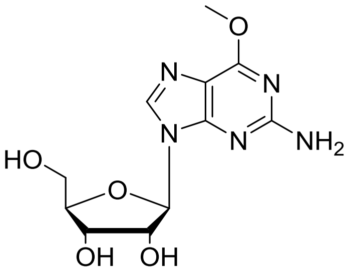

This compound was synthesised according to a modified version of a reported procedure.[8] To a stirred solution of 2', 3', 5'-*O*-tri(*t*-butyldimethylsilyl)-*O*^{6}-methylguanosine (0.6 g, 0.93 mmol) in CH_2Cl_2 was added 3HF.NEt_3 (450 µL, 2.8 mmol) dropwise; the reaction mixture was stirred for 48 hr. The solution was concentrated under reduced pressure. The mixture was purified (99:1 to 9:1 EtOAc / MeOH) by column chromatography which resulted in a white solid (0.2 g, 72 %). TLC R_f 0.30 (9:1 CH_2Cl_2 / MeOH). m.p: 133.7°C. ^{1}H NMR (600 MHz, DMSO-d_6) δ 3.54 (ddd, *J* = 11.5, 5.0, 4.0 Hz, 1H), 3.64 (ddd, *J* = 11.5, 5.0, 4.0 Hz, 1H), 3.90 (q, *J* = 4.0 Hz, 1H), 3.97 (s, 3H), 4.08-4.11 (m, 1H), 4.47 (q, *J* = 6.0 Hz, 1H), 5.08 (t, *J* = 5.0 Hz, 1H), 5.12 (d, *J* = 5.0 Hz, 1H), 5.39 (d, *J* = 5.0 Hz, 1H), 5.79 (d, *J* = 6.0 Hz, 1H),6.44 (s,2H), 8.10 (s, 1H). ^{13}C NMR (151 MHz, DMSO) δ 53.7, 61.9, 70.9, 73.9, 85.7, 87.0, 114.5, 138.4, 154.6, 160.3, 161.2. Calculated for $C_{11}H_{16}O_5N_5$ [M+H]+ 298.1146. Observed: 298.1146.

References

1. W. Aik, M. Demetriades, M. K. K. Hamdan, E. A. L. Bagg, K. K. Yeoh, C. Lejeune, Z. Zhang, M. A. McDonough and C. J. Schofield, *Journal of Medicinal Chemistry*, 2013, 56, 3680-3688.
2. R. J. Hopkinson, R. B. Hamed, N. R. Rose, T. D. Claridge and C. J. Schofield, *Chembiochem*, 2010, 11, 506-510.
3. J. Jeener, B. H. Meier, P. Bachmann and R. R. Ernst, *Journal of Chemical Physics*, 1979, 71, 4546-4553.
4. C. L. Perrin and T. J. Dwyer, *Chemical Reviews*, 1990, 90, 935-967.
5. R. J. Hopkinson, I. K. Leung, T. J. Smart, N. R. Rose, L. Henry, T. D. Claridge and C. J. Schofield, *PLoS One*, 2015, 10, e0145085.
6. N. Usman, K. K. Ogilvie, M. Y. Jiang and R. J. Cedergren, *Journal of the American Chemical Society*, 1987, 109, 7845-7854.
7. K. K. Ogilvie, *Canadian Journal of Chemistry*, 1973, 51, 3799-3807.
8. M. C. Pirrung, S. W. Shuey, D. C. Lever and L. Fallon, *Bioorganic & Medicinal Chemistry Letters*, 1994, 4, 1345-1346.
9. E. Kierzek and R. Kierzek, *Nucleic Acids Research*, 2003, 31, 4461-4471.
10. K. Aritomo, T. Wada and M. Sekine, *Journal of the Chemical Society, Perkin Transactions 1*, 1995, DOI: 10.1039/P19950001837, 1837-1844.
11. H. Tanaka and K. Ogasawara, *Tetrahedron Letters*, 2002, 43, 4417-4420.
12. G. Jia, Y. Fu, X. Zhao, Q. Dai, G. Zheng, Y. Yang, C. Yi, T. Lindahl, T. Pan, Y.-G. Yang and C. He, *Nat Chem Biol*, 2011, 7, 885-887.
13. E. J. Corey and A. Venkateswarlu, *Journal of the American Chemical Society*, 1972, 94, 6190-6191.
14. M. K. Lakshman and J. Frank, *Organic & Biomolecular Chemistry*, 2009, 7, 2933-2940.
